# Mid-IR light modulators enabled by dynamically tunable ultra-high-Q silicon membrane metasurfaces

**DOI:** 10.1038/s41467-026-75121-6

**Published:** 2026-07-17

**Authors:** Felix Ulrich Brikh, Aleksei Ezerskii, Olesia Pashina, Nikita Glebov, Wenhong Yang, Wenping Yin, Sergey V. Makarov, Mihail Petrov, Ivan Sinev, Hatice Altug

**Affiliations:** 1 Institute of Bioengineering, École Polytechnique Fédérale de Lausanne (EPFL), Lausanne, Switzerland; 2 School of Physics and Engineering, ITMO University, St. Petersburg, Russia; 3 Qingdao Innovation and Development Center, Harbin Engineering University, Quingdao, China; 4 University of Brescia, Brescia, Italy

**Keywords:** Metamaterials, Nanophotonics and plasmonics

## Abstract

Metasurfaces enable subwavelength control of free-space light for applications in sensing, nonlinear optics, and quantum photonics. However, their practical deployment is hindered by two key limitations: a tradeoff between low-*Q* resonances and weak amplitude contrast, and their predominantly static nature allowing only passive functionalities. These challenges are particularly severe in the mid-infrared (mid-IR), where the scarcity of low-loss materials constrains performance and scalability. Here, we demonstrate actively tunable single-crystalline silicon membrane metasurfaces combining high-*Q* resonances, strong amplitude contrast, and wafer-scale manufacturability. Our platform achieves record-high measured *Q*-factors up to 3000 in the mid-IR and supports two dynamic modulation schemes: electro-thermal tuning via Joule heating with  >50% modulation depth at CMOS-compatible voltages and speeds up to 14.5 kHz, and ultrafast all-optical modulation via carrier generation in silicon with nanosecond response times and estimated sub-GHz rates. These results establish silicon membrane metasurfaces as a scalable platform for active mid-IR photonics.

## Abstract (web summary)

Mid-infrared metasurfaces have the potential to enable advanced sensing and communication technologies, but scalable active devices remain challenging. Here, the authors demonstrate wafer-scale silicon membrane metasurfaces with record-high quality factor resonances for dynamic light modulation.

## Introduction

Metasurfaces are ultrathin engineered materials composed of sub-wavelength resonators that can manipulate light with unprecedented precision, enabling control over its phase, spectrum, and polarization^1–3^. This unique capability allows them to achieve complex optical functionalities efficiently within a compact footprint, matching and surpassing the performance of classical optical devices^4–9^. Metasurfaces with ultrahigh quality factor resonances attract particular attention, as their narrowband spectral response and concurrent strong field enhancement enable diverse applications such as efficient lasing^10^, high-resolution spectroscopy^11^, enhanced light-matter interactions^12^, and strong nonlinear optical effects^13^. High-quality factors in metasurfaces have been achieved using the concept of bound states in the continuum (BIC) — perfectly localized optical modes^14^. Although an ideal BIC mode is completely decoupled from the continuum of free-space modes, small disturbances, such as symmetry breaking, lead to the introduction of finite radiative losses to the system and the manifestation of sharp resonances in its far-field spectra^15^. In particular, quasi-BIC designs that use unit cell symmetry breaking to fine-tune the radiative coupling have become widely used for precise control of the resonance linewidth^16^.

While high-*Q* resonances strongly enhance the performance of static devices^17^, many emerging applications require dynamic control over the optical response. Actively reconfigurable metasurfaces^18,19^ allow on-demand modulation of resonance frequency, linewidth, or amplitude, dramatically expanding their functional versatility towards adaptive filters^20^, LIDARs^21^, and non-reciprocal optical devices^22^. Various tuning mechanisms have been explored for metasurfaces operating in the visible and near-IR ranges, employing external stimuli such as heat^23^, voltage^24^, photoexcitation^25^, or mechanical deformation^26–28^. Those approaches rely on changing either the intrinsic material properties of the metasurface (phase transitions^20^, carrier concentration^25^, piezoelectric^29^, thermo-optic^30^, electro-optic^31^, or electro-thermal effects) or the environment (alignment of liquid crystals^32^, material control through pressure or chemical reactions^33,34^).

Mid-infrared (mid-IR) photonics have the potential to greatly benefit from both the high *Q*-factor modes and active tuning, which can be utilized for enhanced detection of chemical and biological compounds^11^, spectroscopy, and efficient high harmonic generation^35^. The field has been historically driven by absorption spectroscopy applications and relied on sophisticated bulk-optic instrumentation that compensated for the lack of efficient sources and detectors. Currently, the landscape is rapidly changing due to the maturing of quantum cascade laser technology^36^ and significant progress in mid-IR emission and detection concepts that are expanding possible applications beyond static sensing and toward dynamic imaging^37^, adaptive spectroscopy, active thermal emission control^38^, and more. These emerging applications require the same level of optical control that is routine in the visible and near-IR (telecom) ranges, such as fast intensity and phase modulation, spatial light modulation, beam steering, and dynamically tunable, CMOS-compatible active components with high spectral selectivity. Notably, despite the significantly relaxed fabrication requirements for mid-IR metasurfaces as compared to their visible/near-IR analogues, experimentally reported *Q*-factors are still moderate and do not reach above a few hundred for either passive or active solutions. This is mostly related to the fact that standard fabrication bases, such as silicon-on-insulator and silicon-on-sapphire that allow *Q*-factors as high as 10^5^ in the telecom spectral range^39^, cannot be scaled towards the mid-IR due to the onset of absorption in the low-index substrate material. At the same time, the commonly used germanium on calcium fluoride platform suffers from grain formation in the sputtered germanium that contributes to the effective non-radiative losses^9^, limiting the achievable quality factors to *Q* ≈ 200. Very recently, free-standing silicon membrane metasurfaces were explored in the mid-IR as a route to higher Q-factors, as they eliminate lossy substrates and reduce non-radiative losses^40,41^. These membrane structures have primarily been studied in the context of surface-enhanced infrared absorption (SEIRA) and strong light-matter coupling and involved fully static operation or slow thermal tuning.

From the tunability perspective, reconfigurable metasurfaces based on chalcogenide phase-change materials enable strong modulation depth but are limited to low switching speeds, as their tuning mechanism relies on heating and cooling sequences^42–45^. Similar restrictions arise in devices relying on gas absorption^46^, intercalation of ions^47^ or thermal tuning^48,49^. Electrostatic tuning of free-carrier-based platforms including graphene^50,51^, ITO^52^, and carbon nanotube films and metamaterials^53–55^ can potentially provide high (sub-GHz) modulation frequencies. However, these approaches rely on plasmonic or intraband responses associated with increased optical losses and typically require large carrier densities to achieve substantial modulation depth, which often translates into elevated operating voltages and reduced spectral selectivity^56^, complicating CMOS-level integration. This highlights that translating the well-developed tuning mechanisms for light control into the mid-IR is not a straightforward wavelength scaling task. Therefore, the development of alternative material platforms and design strategies for metasurfaces that can simultaneously support high-*Q* resonances and efficient, high-speed, low-power tunability in the mid-IR, while maintaining compatibility with wafer-scale manufacturing, is critically important for practical applications.

**Fig. 1:**
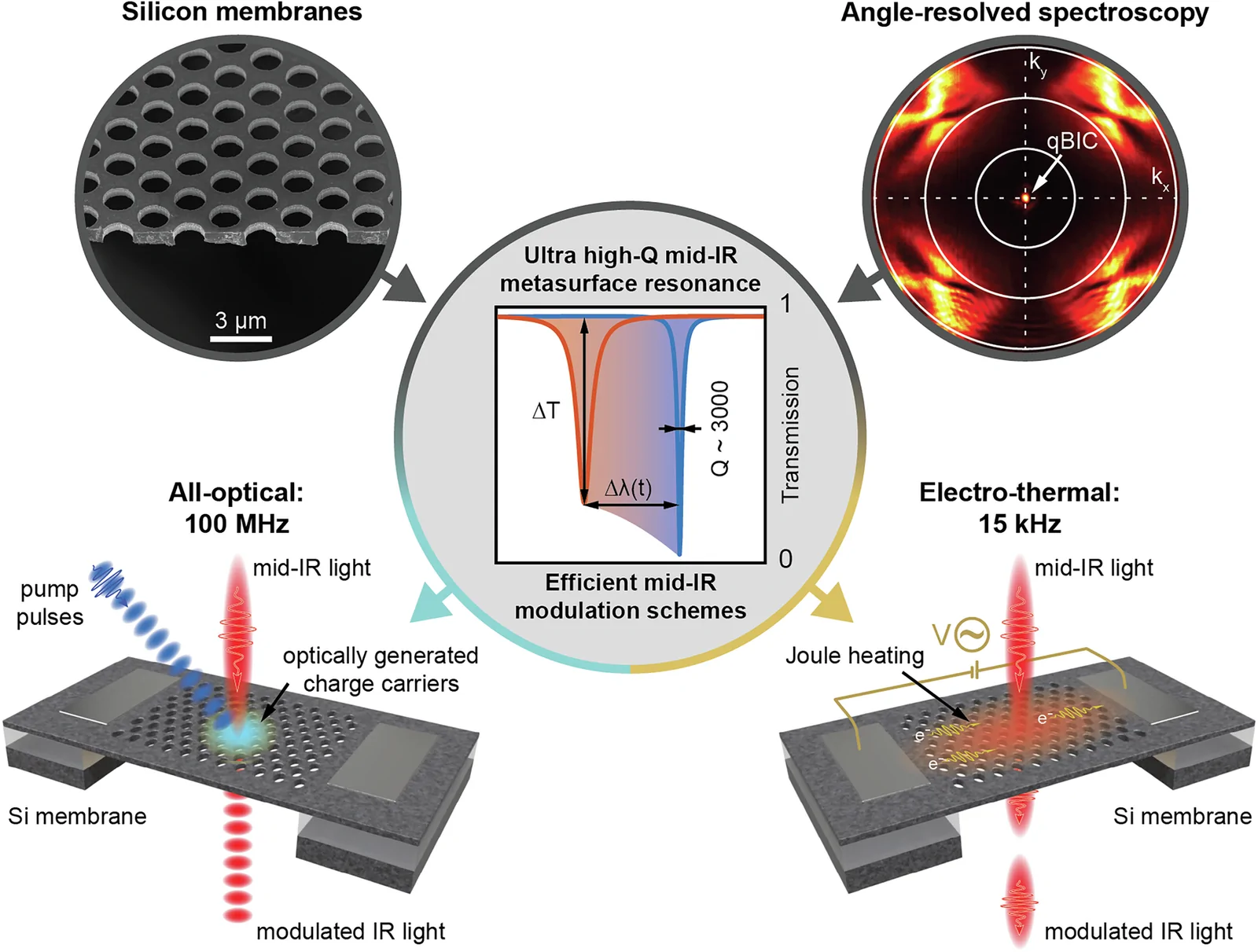
Mid-IR metasurface modulators. Crystalline silicon membrane metasurfaces (top left) in combination with angle-resolved spectroscopy measurements (top right) allow to resolve large amplitude, ultra-high quality factor resonances in mid-IR (middle). This enables efficient mid-IR light modulation schemes. Bottom left: high-speed modulation with an optical pump that induces a refractive index change through the generation of non-equilibrium charge-carrier density. Bottom right: electro-thermal modulation by direct Joule heating of the membrane at CMOS-compatible applied voltages.

In this work, we introduce dynamically tunable high-*Q* mid-IR metasurfaces based on single-crystalline silicon membranes. We achieve record-high experimentally measured quality factors (*Q* up to 3000) for free-space mid-IR photonic structures. The extremely narrow resonance linewidth, combined with large (>50%) absolute amplitude, enables efficient modulation of the metasurface optical response for moderate external drive. We showcase two realizations of actively controlled mid-IR signal modulation (Fig. 1). In the first one, we demonstrate an on-chip device that features strong electro-thermal modulation induced by a CMOS-compatible voltage of 5 V sustaining >50% absolute signal variation with frequencies up to 14.5 kHz. This platform with integrated, actively modulated heaters enables significantly faster and more precise control compared to the external thermal heating methods^48^. In the second, we achieve high-speed (sub-GHz) all-optical modulation using the ultrafast optically induced charge-carrier generation in silicon. Sharp resonances combined with the strong sensitivity of the optical response of silicon to the generated charge density in the mid-IR range allow for efficient modulation at pump fluences nearly two orders of magnitude lower than for similar solutions in the near-IR and visible spectral ranges^57^. Importantly, we demonstrate full compatibility of the design with wafer-scale manufacturing using deep-UV lithography (DUVL) without compromising optical performance compared to chip-scale electron beam lithography. Our results establish membrane metasurfaces as a versatile, scalable platform for high-speed, high-contrast, low-power active light routing in the mid-IR, where efficient and actively tunable free-space photonic devices remain very scarce^58^. We envision applications of our platform in free space communications, thermal emission management, chemical and biological sensing, as well as in the emerging fields of mid-IR quantum optics.

## Results

### Ultra high-*Q* resonances in membrane metasurfaces

**Fig. 2:**
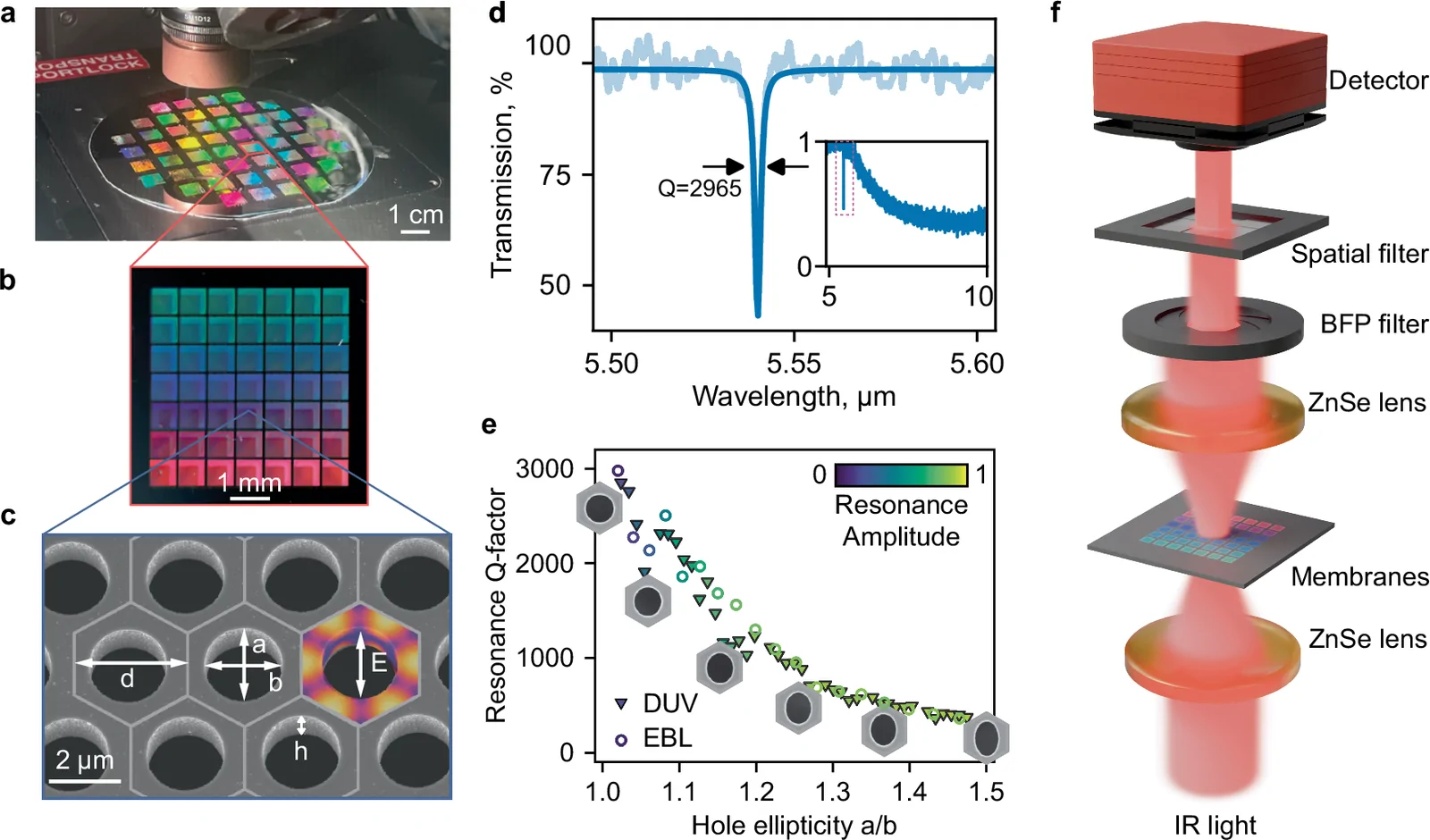
Achieving ultra-high quality factors in mid-IR metasurfaces. **a** Image of wafer-scale membranes. **b** Image of a single 1 × 1 cm^2^ chip with 7 × 7 metasurfaces. **c** SEM image of a DUV-fabricated Si membrane metasurface. Colorful overlay on one of the unit cells shows the calculated distribution of the electric field at the qBIC resonance excited with linearly polarized plane wave **E** at *λ*_res_ = 5.54 μm. **d** Transmission spectrum of the membrane metasurface with the measured resonance *Q*-factor of ~2965 and high amplitude contrast. The inset shows the same spectrum in a broader wavelength range. **e** Dependence of the measured *Q*-factor for EBL (hollow circles) and DUV (solid triangles) written membrane metasurfaces with varying hole ellipticity. The color of the symbols encodes the measured resonance amplitude. The insets show SEM images of the corresponding unit cell. **f** Schematic of the mid-IR spectroscopy setup for high-*Q* resonance measurements.

Our membrane metasurfaces are based on CMOS-grade silicon-on-insulator substrates that are compatible with high-throughput wafer-scale processes. To incorporate a high-Q mode with a linewidth that can be easily controlled with design parameters, we utilize the well-established concept of symmetry-protected bound states in the continuum^59^. As a base design, we choose the highly symmetric hexagonal (C_6*v*_) lattice of circular holes in a silicon slab to ensure robustness of the resulting suspended membrane. The non-radiative BIC states supported in such a lattice can be transformed into modes with finite *Q*-factors by breaking the symmetry using the quasi-BIC principle^16^ according to the established selection rules^60^. In our case, we reduce the symmetry of the structure to C_2*v*_ by introducing ellipticity to the holes: $\varepsilon=\frac{a}{b}$ (Fig. 2c).

Using this design principle, we numerically optimize the structure parameters to rescale the BIC condition to the 5–6 μm spectral range using the recent paper^61^ as a reference. Fixing the design to a commercially available base silicon layer thickness of *h* = 1 μm, we end up with a structure with a lattice period of *d* = 3.3 μm. To tune the *Q*-factor without a considerable spectral shift of the resonance, when changing the ellipticity, we keep the hole area constant at *A* = 3.5 μm^2^.

Unlike other designs with small features or multiple resonators per unit cell, our metasurfaces feature large critical dimensions (~1 μm) that make them fabrication-friendly, robust to imperfections, and compatible with high-throughput, wafer-scale techniques. Here, we demonstrate the compatibility of our design with both chip-scale e-beam lithography and wafer-scale deep-UV (DUV) lithography (Fig. 2).

The crystallinity of the metasurface material and the absence of a substrate that can induce additional optical losses make the silicon membrane-based metasurfaces ideal for achieving ultra-high *Q*-factors in the mid-IR range. However, direct observation of these high *Q*-factors is also largely dependent on the measurement procedure, as the linewidth and spectral position of the qBIC modes tend to change rapidly with the incidence angle. As a result, standard FTIR microscopes with reflective Cassegrain objectives or high NA refractive objectives are not well suited for characterization of high-*Q* modes. For accurate measurements of our membrane, we developed a custom FTIR microscope accessory with spatial filtering of optical signal in the back focal plane, as illustrated in Fig. 2f. By closing the size of the filter aperture down to 1 mm we reduced the effective NA of the optical system down to 0.02, which allowed us to isolate the response from near-normal angles of incidence that correspond to the highest mode Q-factors. This NA corresponds to a full angular divergence of approximately 2.3^°^ (40 mrad), which complies with typical mid-IR beam sizes and their divergence. Figure 2d shows the transmission spectrum of the metasurface with a hole ellipticity of *ε* = 1.08 fabricated with e-beam lithography (EBL). The spectrum features an extremely sharp resonance dip at around *λ *= 5.54 μm. Notably, it combines a very large amplitude contrast close to 55% and a high quality factor of *Q* = 2965, which are equally important for both sensing and light modulation applications^62^. This represents a major improvement over the previously reported mid-IR metasurfaces, including germanium on a CaF_2_ platform^63^ as well as alternative membrane designs^41^. By changing the hole ellipticity from *ε* = 1 to 1.5, the *Q*-factors can be tuned in a broad range from several hundreds up to a maximum value of *Q* ≈ 3000 as shown in Fig. 2e. Higher quality factors exhibit smaller modulation depths, which is limited by the residual fabrication imperfections. Structures fabricated with DUV (data marked with triangles) show *Q*-factors measured at up to 2835, comparable to the EBL (data marked with hollow circles), with slightly smaller resonance amplitude due to reduced fabrication fidelity.

**Fig. 3:**
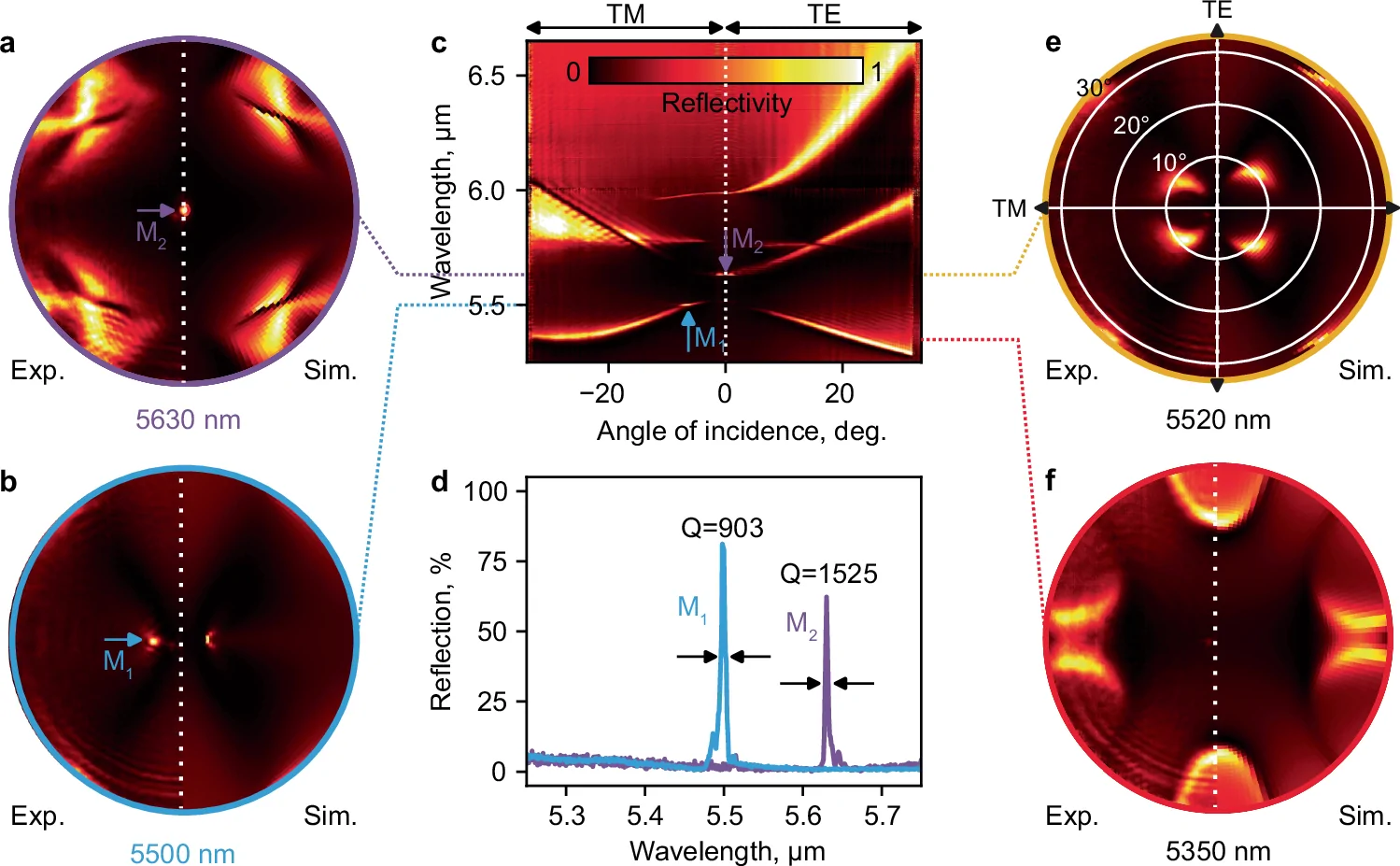
Angular and polarization dispersion of membrane metasurfaces. **a**, **b** Back focal plane images of the membrane metasurface at two wavelengths (5.63 and 5.5 μm) corresponding to its two high-*Q* resonances (M_1_, M_2_). The left part of each panel shows experimental reflectivity measured with an open optics QCL system. The right part shows the simulation data for angle and polarization-resolved reflectivity. **c** Map of angle-resolved reflectivity for TE and TM polarization extracted from the experimentally measured back focal plane images. High-*Q* modes M_1_ and M_2_ are indicated with arrows. **d** Metasurface reflectivity resonances extracted from the measured back focal plane data. The *Q*-factors are modified with respect to the extracted values from customized FTIR measurements due to the finite imaging quality of the BFP setup. **e**, **f** Back focal plane images of the membrane metasurface for off-resonant wavelengths (5.52 and 5.35 μm). The overlay in panel (**e**) shows the polar axes common for images in (**a**, **b**, **e**, **f**).

To further characterize the optical properties of high-*Q* membrane metasurfaces, we developed a mid-IR back focal plane (BFP) open optics imaging setup (see “Methods” section for more details). It is based on a quantum cascade laser assembly (from Daylight Solution Spero) that provides a narrow (<0.01 cm^−1^) emission line tunable in a broad spectral range (5–10 μm). In the setup, we focus the laser radiation on the sample surface through a 0.56 NA black-diamond lens, accommodating incident angles up to  ~34^°^. We then record the reflectivity images of the back focal plane of the system, projected on a mid-IR microbolometer camera (DataRay IR-BB) using a 4*f* lens assembly. Collecting these images while tuning the laser wavelength, we acquire polarization- and angle-resolved hyperspectral reflectivity data that manifest the isofrequency curves of the metasurface modes. Four exemplary back focal plane images (normalized to reflection images from a gold mirror) are presented in Fig. 3a, b, e, f (see Supplementary Video 1 for full spectral dependence and comparison with simulation). The right half of each plot is replaced with the corresponding BFP data obtained with numerical simulations and shows perfect agreement with the experiments. In particular, Fig. 3a, b show the BFP images at wavelengths close to the high-Q resonances of the metasurface, which feature sharp peaks with extremely narrow angular spread.

We further process the hyperspectral BFP data to extract the dispersion of the optical modes of our metasurface. Figure 3c shows the reflectivity maps for pure TE and TM polarization of the incident light, obtained by stacking the vertical and horizontal middle sections of each recorded BFP image. Two dispersive high-*Q* modes (marked M_1_ and M_2_) are clearly visible, with M_2_ corresponding to the qBIC mode that strongly manifests for TE polarization. Vertical sections in Fig. 3c represent the reflectivity spectra at selected incidence angles, while Fig. 3d highlights the spectra of modes M_1_ and M_2_. From this data, we extracted *Q*-factors of *Q*_1_ = 903 and *Q*_2_ = 1525. The smaller *Q*-factors, compared to measurements with the customized FTIR, can be attributed to the aberrations and limited imaging performance of our BFP setup. These effects reduce the measured *Q*-factors by broadening the detected peaks, analogous to averaging over a finite angular range.

After establishing silicon membrane metasurfaces as a platform supporting ultra-high-*Q* resonances in the mid-IR, we leverage them to achieve active mid-IR light modulation with high contrast in two complementary ways. For this, we rely on the outstanding properties of silicon: firstly, its high thermo-optical coefficient for on-chip electro-thermal tuning and secondly, the strong dependence of its refractive index on the charge carrier density for fast all-optical tuning.

### Electro-thermal modulation

**Fig. 4:**
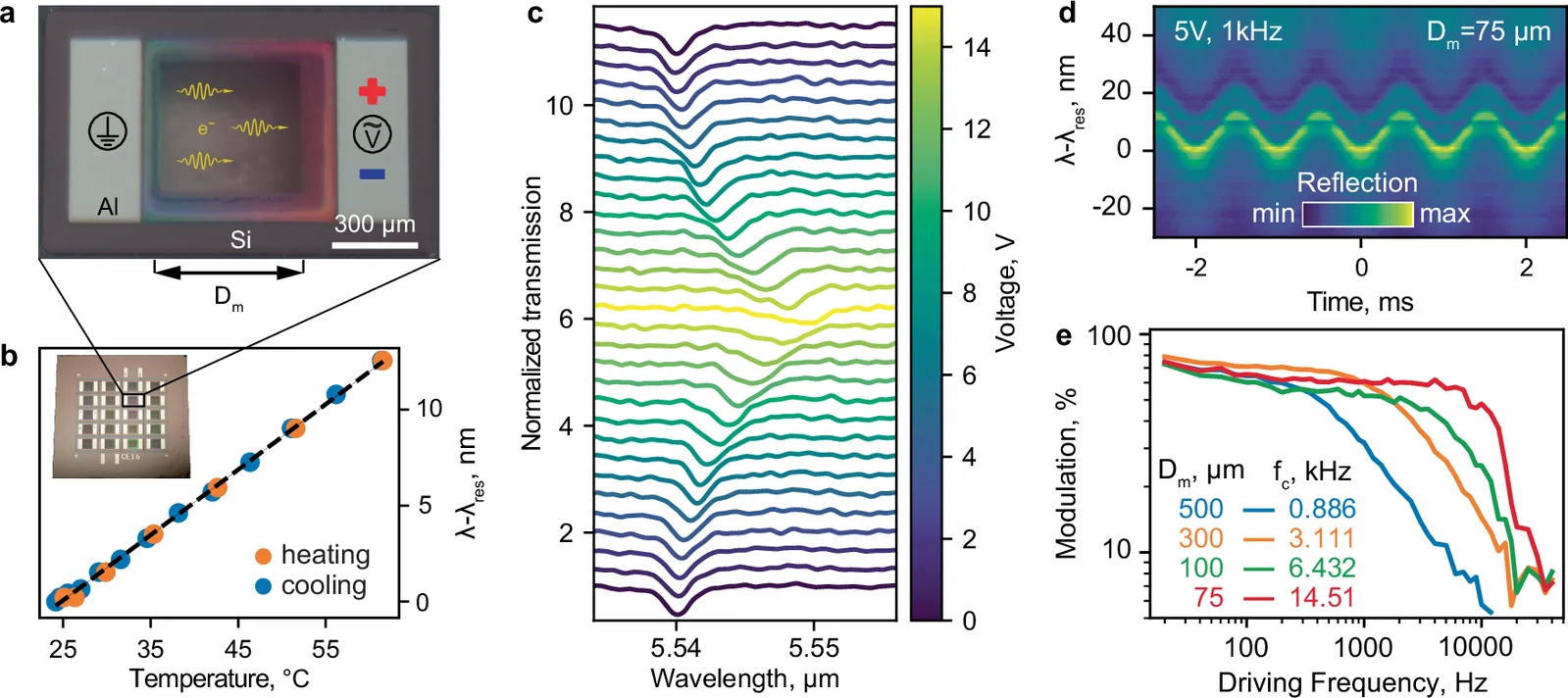
Electro-thermal modulation. **a** Image of the mid-IR electro-optical modulator, consisting of a membrane metasurface with a size D_m_ and two aluminum electrodes. The overlay visualizes the Joule heating induced by the current passing through the membrane. **b** Dependence of the measured resonance wavelength shift on the membrane temperature. The inset shows an image of a 9 × 9 mm^2^ chip featuring 20 electro-thermally tunable devices. **c** Dependence of a 500 μm metasurface transmission spectra on the applied static voltage (encoded by the color of each curve). **d** Modulation of the spectral position of the metasurface reflectivity resonance recorded in the time domain with the back focal plane imaging setup. **e** Amplitude-frequency characteristics for metasurface modulators with different membrane sizes *D*_*m*_ measured at 5 V driving voltage. The inset shows the cutoff frequency *f*_*c*_ for each case.

To realize electro-thermal modulation, we implement direct Joule heating of the membrane by passing an electrical current through it. For this purpose, we evaporate thin-film aluminum electrodes onto the opposite sides of the metasurface, thus enabling the current flow with minimal perturbation of the optical response (see Fig. 4a). To facilitate sufficient heating efficiency at CMOS-compatible voltages, we lightly dope the silicon membrane with phosphorus (see “Methods” for details). This introduces a trade-off: while doping reduces the electrical resistance and enables more efficient heating at low voltages, it also increases the non-radiative optical losses, eventually deteriorating the resonance quality factor. The doping level was therefore carefully optimized to balance the electrical conductivity with optical performance (see Supplementary Notes 2, 3).

High quality factor and large resonance amplitude of our membrane metasurfaces lead to efficient tuning, as the relatively high thermo-optical coefficient of silicon still measures at only 2 ⋅ 10^−4^ K^−1^ (see Supplementary Note 1). Therefore, the extremely narrow resonance linewidth facilitates a strong modulation depth for minimal temperature changes. This not only increases the tuning efficiency but is also critical for reaching higher modulation speeds, for which the heat dissipation becomes the main challenge.

To calibrate the electro-thermal tuning, we first performed the spectroscopy experiments with external heating of the metasurface using a ceramic hotplate in a temperature range of 25–60^°^C (Fig. 4b). We then applied a series of static voltages across the metasurface and recorded the corresponding transmission spectra. The results reveal a clear shift in the resonance with an increase in the applied voltage (Fig. 4c). The shift scales linearly with the temperature (Fig. 4b) and quadratically with the applied voltage (see Supplementary Figure 1d), consistent with the expected linear relationship between the resonance wavelength and the heating power. Remarkably, a temperature change of only 35^°^C leads to a resonance shift of up to 5 FWHM (≈9 nm for *Q* = 2965 and *λ*_res_ = 5.54 μm) and an absolute modulation amplitude of 70%.

We then studied the dynamic performance of the electro-thermal tuning mechanism by modulating the applied voltage. The long acquisition time of FTIR spectra prevents the observation of fast resonance tuning. Therefore, we used our open optics setup with QCL excitation (see “Methods” for details) and applied a spatial filter in the Fourier plane of the optical system to isolate the response of the high-*Q* resonance mode. In the experiment, the voltage-induced refractive index change steers the metasurface reflectivity maximum away from the aperture of the spatial filter. The corresponding signal change is then measured with a fast mercury cadmium telluride (MCT) detector. By scanning the excitation wavelength and tracking the detector readout with an oscilloscope, we can track the temporal modulation of the metasurface resonance Fig. 4d. With a driving voltage of 5 V at 500 Hz, the resonance clearly follows the sinusoidal curve with a frequency of 1 kHz, as expected from the quadratic dependence of dissipated power on voltage.

As mentioned above, the main limiting factor for electrically driven suspended membrane systems is the slow heat dissipation due to the poor thermal conductivity of air. The restricted thermal flow hinders the cooling rate and, consequently, the modulation speed. We address this issue by fabricating smaller membranes, which have more efficient lateral heat dissipation. However, when reducing the membrane size, one needs to keep in mind that the BIC mode used in our design is of a collective nature and requires a minimum number of unit cells to maintain its ultra-high *Q*-factor and resonance amplitude. Earlier studies on this subject reported the lateral spread of qBIC modes within up to 20 unit cells^64–66^. In our case, we achieve ten-fold higher *Q*-factors, indicative of even larger mode volumes. Based on this analysis, we fabricated metasurface membranes with lateral sizes of *D*_m_ = 500; 300; 100; 75 μm, and studied their amplitude-frequency response (see Supplementary Note 4). Figure 4e shows that all of the metasurfaces exhibit a base modulation depth of 70%, confirming that their size is sufficient to support an unperturbed high-*Q* optical mode. At the same time, the reduction of the membrane size drastically improves the cutoff frequency due to more efficient lateral heat dissipation that allows faster membrane recovery (see Supplementary Note 5). The maximum electro-thermal modulation speed reached 14.5 kHz for the *D*_m_ = 75 μm membrane, with the total dissipated power of 32 mW.

### All-optical modulation

To achieve fast modulation of mid-IR light, we utilized optically induced charge-carrier generation. This mechanism was previously successfully used for tuning the optical response of silicon photonic structures in the visible^25,67^. The mid-IR range is particularly interesting for this all-optical tuning approach, as the increase of the charge carrier density blue-shifts the silicon plasma frequency close to the spectral range of the resonance. This leads to a strong modulation of the refractive index at moderate charge densities, minimizing the power consumption. Here, we achieve strong modulation for laser fluences less than 5 μJ cm^−2^, almost 2 orders of magnitude lower than what is required for visible and near-IR applications^57,67,68^

**Fig. 5:**
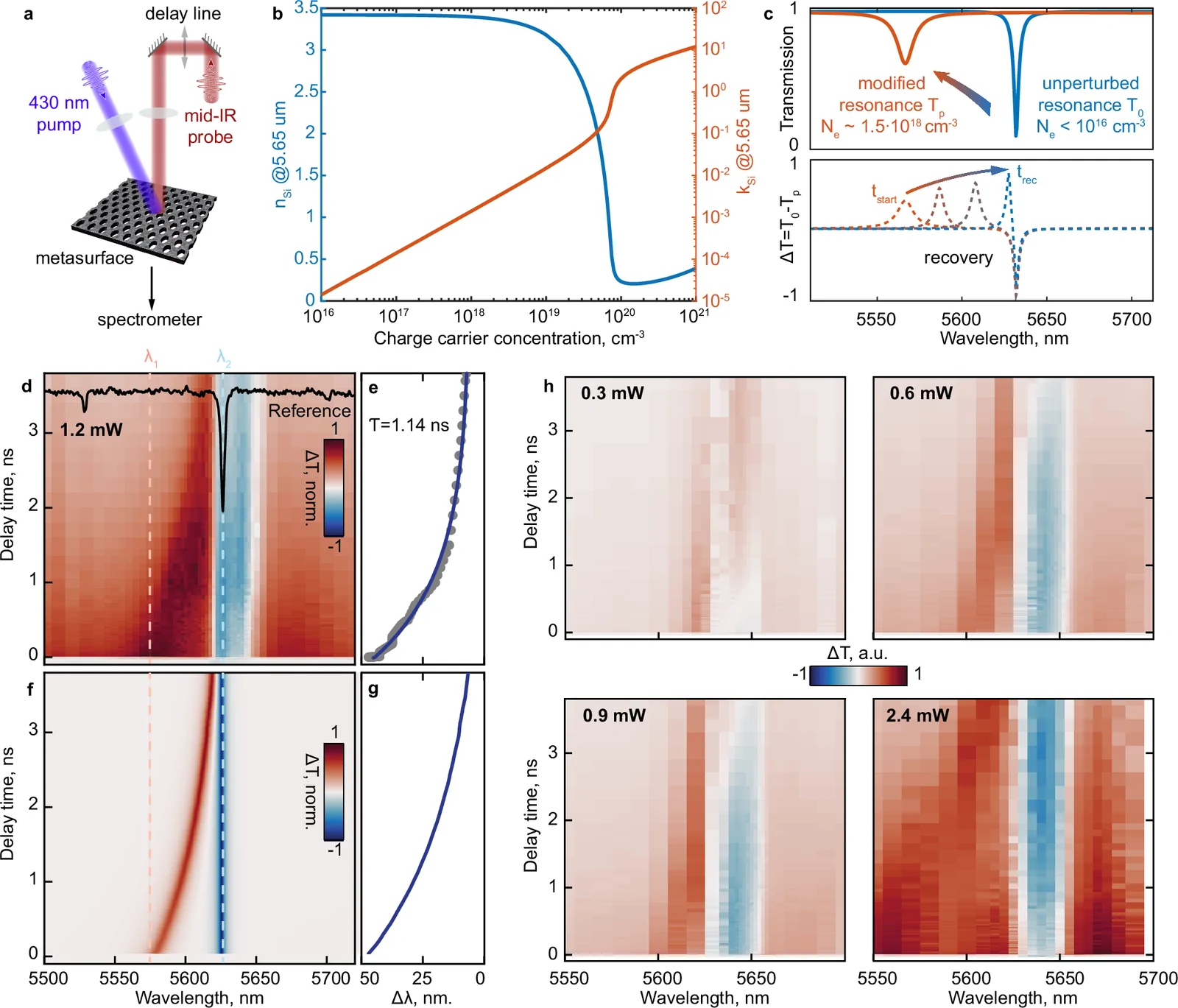
Ultrafast all-optical modulation. **a** Schematic of the pump-probe experiment for studying the temporal dynamics of membrane metasurfaces. **b** Calculated dependence of the real and imaginary parts of the refractive index of crystalline silicon at *λ *= 5.65 μm on the charge carrier density *N*_*e*_. **c** Illustration of the all-optical tuning concept. Top: the narrow transmission resonance of the unperturbed metasurface (blue curve) is blue-shifted and broadened (red curve) when the incoming pump pulse induces the non-equilibrium charge carrier density. In the spectrum of differential transmission *Δ*T (bottom), this leads to the formation of a characteristic asymmetric profile with a dip at the original resonance position, which gradually recovers over time. **d** Temporal dynamics of the normalized differential transmission of the membrane metasurface for a pump-probe delay time range of 0–3.6 ns. The metasurface is pumped optically at *τ *= 0 with a 430 nm, 290 fs pulse at 10 kHz repetition rate with an average power of 1.2 mW. The black curve in the overlay shows the transmission spectrum of the unperturbed metasurfaces measured with FTIR. **e** Temporal evolution of the resonance shift. The dots show data points extracted from the experiment, the curve is the fitting of data with a single exponent with *τ *= 1.14 ns. **f**, **g** Time-resolved dynamics of the metasurface resonance calculated assuming a decay time of *N*_*e*_ of 2.28 ns. **h** Measured time-resolved differential transmission of the metasurface under different pump powers is indicated in each panel.

In our experiments, we employed visible light pulses (*λ* = 430 nm) generated in the mid-infrared transient absorption spectroscopy system (Light Conversion HARPIA-TA, see “Methods”) to generate non-equilibrium charge carriers in the silicon membrane, thus altering its refractive index in an ultrafast manner. We then probed the modifications to the metasurface response using a spectrally tunable mid-infrared probe pulse with a controlled time delay with the pump (Fig. 5a). We estimated the expected refractive index change with simulations of the charge-density-dependent properties of silicon, taking into account the plasma frequency shift, band-filling effect, and changes to the band gap. The simulation results for the real and imaginary parts of the refractive index at the resonant frequency of the metasurface (*λ *= 5.65 μm) are shown in Fig. 5b. They reveal that the real part *n* exhibits a gradual decrease with the increase of *N*_*e*_, accompanied by a steady growth of loss *k*. At a moderate charge density of *N*_*e*_ = 7.4 ⋅ 10^−19^ cm^−3^, silicon is metallized in this spectral range as its permittivity drops below zero.

The concept of ultrafast all-optical modulation of the high-*Q* membrane metasurface is shown in Fig. 5c. The spectrum of the unperturbed metasurface (*T*_0_) exhibits a sharp transmission dip (blue curve). With the arrival of the pump pulse, the generated charge carriers decrease the refractive index of the membrane material (Fig. 5b), which leads to a spectral blueshift of the resonance (*T*_*p*_). In the differential transmission spectrum *ΔT* = *T*_0_ − *T*_*p*_ (dashed curves), this manifests itself as a bisignate profile, which then merges together during the subsequent charge recombination process (decay of *N*_*e*_).

The charge recombination time is a critical parameter that defines the membrane recovery time. Bulk crystalline silicon has low defect density, which extends the charge lifetime. Our simulations (see Supplementary Note 6) indicate that the surface recombination process dominates the charge decay at the estimated peak concentration values, while the Auger process becomes relevant for concentrations of at least an order of magnitude larger. Furthermore, in the experiment, patterning the membrane significantly increases the surface area, further facilitating the corresponding charge recombination mechanism. We measure the decay time directly in pump-probe experiments by using a long (4 ns) delay line and tracking the recovery of the metasurface resonance in differential transmission spectra. Fig. 5d shows the map of *ΔT* measured for the average pump power of 1.2 mW (equivalent laser fluence 4.8 μJ cm^−2^). As expected, the arrival of the pump pulse at (probe delay *τ *= 0) induces a characteristic bisignate pattern in the spectrum, with a dip at the wavelength of the unperturbed resonance and a blueshifted peak corresponding to the optically modified mode (see also illustration in Fig. 5c). Note that the peaks in the probe pulse transmission spectra are broadened as compared to linear spectroscopy data, which is shown with a black curve in Fig. 5d. This is due to the divergence of the focused probe beam, leading to the averaging of the response over multiple angles. Increasing *τ* reveals a gradual recovery of the transmission profile governed by the decay of *N*_*e*_. The metasurface resonance shifts from the maximally detuned position *λ*_1_ to the unperturbed value *λ*_2_, both marked with vertical dashed lines in the map. From these data, we extracted the dependence of the resonance peak shift on the probe delay time, which is shown in Fig. 5e. Fitting the data with a single exponent yields a maximum detuning of 50 nm and the characteristic decay time of the resonance shift of 1.14 ns. As the resonance shift has an almost linear dependence on the refractive index and, consequently, a square-root dependence on the concentration *Ne*, we estimate the carrier-concentration decay time to be 2.28 ns. We then performed numerical simulations of the time-resolved metasurface transmission spectra for a broad range of concentration values using the data from Fig. 5b. For each time delay value, we retrieved the corresponding charge concentration by fitting the calculated resonance wavelength to the transient transmission maxima extracted from the experiment (Fig. 5e). The fitting yielded a maximum concentration of *N*_*e*_ = 1.5 ⋅ 10^18^ cm^−3^ and a lifetime of 2.25 ns consistent with the fitting of the resonant shift lifetime (Fig. 5f, g). According to our estimations, this is fully determined by a rather high surface recombination velocity (5 ⋅ 10^4^ cms^−1^), assisted by the membrane patterning, while Auger processes are expected to manifest for at least an order of magnitude higher initial concentrations. The transient transmission spectra measured for different pump powers (Fig. 5h) indicate that lower pump powers naturally lead to smaller initial resonance shifts, with the bisignate lineshape almost vanishing for the pump power of 0.6 mW. For the maximum available pump power of 2.4 mW, we observed resonance detunings of up to 80 nm, which corresponds to the induced carrier concentrations of  ≈ 5 ⋅ 10^18^ cm^−3^.

The short decay time of the carrier concentration facilitates high modulation speeds. To estimate their limits, we consider both the temperature stability of the membrane and the charge relaxation. The data on electro-thermal tuning indicated that the metasurface could sustain dissipation of at least 32 mW of power. For the membrane size of 75 μm, which is able to support the high-*Q* resonance, this dissipated power is equivalent to a complete absorption of the 4.8 μJ cm^−2^ laser pump pulse at a repetition rate of  ≈ 120 MHz. On the other hand, at this modulation frequency, the resonance shift *Δλ* decays down to 2 nm before the arrival of the next pulse. This puts the detuning below the resonance FWHM, indicating sufficient recovery of the transmission amplitude.

## Discussion

We have demonstrated the vast potential of single-crystalline silicon membrane metasurfaces as a versatile platform for active mid-IR photonics. The crystalline material quality, combined with the absence of a lossy substrate, enables record-high measured resonance quality factors up to 3000, over an order of magnitude higher than previously reported metasurfaces based on amorphous materials (see Supplementary Note 7). The narrow resonance linewidth, together with strong amplitude contrast and fabrication-friendly critical dimensions, provides the foundation for dynamic and scalable mid-IR photonic devices.

Building on this platform, we implemented two complementary modulation schemes. Electro-thermal tuning achieves large modulation depths exceeding 50% at CMOS-compatible voltages, while scaling to modulation speeds of up to 14.5 kHz through membrane size optimization. In parallel, all-optical modulation *via* laser-induced charge carrier generation provides nanosecond response times, supporting estimated sub-GHz operation at low pump fluences. These approaches overcome the trade-off between large modulation amplitude and high modulation speeds.

By uniting high resonance *Q*-factors with strong amplitude contrast, efficient dual-mode tunability and wafer-scale fabrication, our silicon membrane metasurfaces address the important bottlenecks in mid-IR photonics technologies. This paves the way for CMOS-compatible, active devices for applications in high-speed and low-power free-space communications, dynamic control of thermal emission, as well as chemical and biological sensing with enhanced spectral selectivity and sensitivity. In quantum optics, the combination of high-Q resonances with both electrical and optical tunability can enable applications such as cavity-enhanced mid-IR single-photon sources, on-chip quantum spectroscopy of molecular vibrations, and tunable generation of entangled photon pairs. Furthermore, our developed platform opens new avenues for future advances in dynamically reconfigurable photonic systems.

## Methods

### Device fabrication

Silicon (Si) membrane metasurfaces were fabricated starting from silicon on insulator (SOI) wafers: 1 μm device Si (boron doped 8.5 Ω ⋅ cm to 11.5 Ω ⋅ cm) - 1 μm silicon dioxide (SiO_2_) buried oxide - 250 μm handle Si. First, the backside openings were defined lithographically in a 3 μm SiO_2_ layer (Plasma-Therm Corial D250L PECVD) using direct laser writing (DLW) (Heidelberg Instruments Maskless Aligner MLA 150, AZ ECI 3027, 3000 rpm) and fluorine-based deep reactive ion etching (DRIE, SPTS Advanced Plasma System). Second, the device Si layer was patterned with a single-step lithography process (DUV: ASML PAS 5500/350C with dose 9.5 mJ/cm^2^, M108Y resist, 2000 rpm; **or** EBL: Raith EBPG5000 with dose 280 μJ/cm^2^, ZEP520 50%, 2000 rpm) followed by DRIE (Alcatel AMS 200 SE, 1500 W SF_6_/C_4_F_8_ plasma at 25/55 sccm flow). Third, the membranes were opened by etching the handle silicon through the previously defined SiO_2_ mask using a DRIE Bosch process (Alcatel AMS 200 SE). Lastly, the buried oxide layer was removed from the opening areas by hydrofluoric acid (HF) vapor etching (SPTS μEtch).

#### Doping

For electro-thermal modulation experiments, the device silicon was doped with phosphorus in the vapor phase (Centrotherm furnace, 1 min to 4 min POCl_3_ at 800  ^°^C, HF deglazing, 30 min annealing at 900  ^°^C). The achieved sheet resistance range of 44.8 Ω/□ to 347 Ω/□ was enough to find a suitable trade-off between optical and electrical properties. The contact electrodes were defined in photoresist (AZ ECI 3027, 3000 rpm) by direct laser writing (*λ* = 405 nm, 200 mJ cm^−2^). After development (AZ 726 MIF, 2 min) and descumming in O_2_ plasma (500 W, 1 min), the native SiO_2_ was stripped by 10 s dip in 7:1 buffered HF, followed by electron beam evaporation (Alliance-Concept EVA 760) of 20 nm titanium and 150 nm aluminum. The final step was the lift-off of the superfluous metal (MICROPOSIT Remover 1165).

### Infrared spectroscopy

#### FTIR Microscope

We obtained the infrared (IR) transmission spectra at normal incidence using a Bruker Vertex 80v FT-spectrometer with an attached IR Microscope (HYPERION 3000) equipped with a liquid nitrogen cooled MCT detector. The metasurfaces were excited using a ZnSe lens with a focal length of 50 mm, mildly focusing linearly polarized IR light on the sample surface. Transmitted light was collected with another 25 mm lens equipped with an additional iris placed at its back focal plane. Closing the iris allowed for limiting the numerical aperture of the system down to approximately 0.02 and thus suppressing the unwanted signal from oblique excitation angles. Signal collection area was limited to an approximately 300 μm square central region of the membrane by a double-blade aperture placed in the conjugate image plane of the IR microscope. The sample chamber was constantly purged with dry air to provide a stable, low level of humidity.

#### Free space BFP setup

The light source for the back focal plane measurement is a tunable QCL laser (Daylight Solutions SPERO QTX 340). The light was focused onto the metasurface using a black-diamond lens (f = 5.95 mm, NA = 0.56). The reflected light was collected and spatially filtered in the real and Fourier planes of a 4*f* system. The signal was then either imaged on a microbolometer-based camera with 600 × 480 pixels (DataRay WinCamD-IR-BB) or detected with an MCT photodiode (Thorlabs, PDAVJ10).

#### Pump-probe measurements

Ultrafast dynamic measurements were conducted using a pump-probe system (Harpia-TA, Light Conversion) with a 4 ns delay line. The femtosecond laser pulses (1030 nm, 200 fs, 50 kHz), generated by the Pharos PH2-20 W laser system (Light Conversion), were split into the pump and probe beams. Each beam was individually tuned using an optical parametric amplifier (Orpheus-HP for the pump and Orpheus-One-HP for the probe). Both beams were focused on the sample, which was positioned orthogonally to the probe beam. Infrared transmission spectra were collected using an Andor KYMERA-193i-B2 spectrograph, equipped with a 150 mm^−1^ mid-IR diffraction grating and a point MCT detector (Infrared Associates MCT-13-1.0). For the average (chopped) measured pump power of 1.2 mW and an approximate beam area of 1 mm^2^, we estimate a fluence of 4.8 μJ cm^−2^.

### Data processing

#### FTIR

Spectral data from the FTIR, normalized to the signal without the sample with the same optics, were fitted with a Fano lineshape to obtain resonance frequency, *Q*-factor and amplitude.

#### IR camera

Back focal plane reflectivity data from the 2D bolometric detector were normalized to the reflection BFP image of a gold mirror for each excitation wavelength.

#### Photodiode

The tunable QCL provides pulses of 20 ns to 500 ns length at a repetition rate of up to 100 kHz, resulting in a maximum duty cycle of 5%. The pulse train was collected with an oscilloscope (Agilent DSO-X 3012A). The oscilloscope sampling rate was set between 10 MSa/sec to 400 MSa/sec, which allowed to resolve each pulse with a minimum of 5 samples while simultaneously recording down to 10 Hz modulation. The pulse train was converted to intensity (Fig. 4d) by averaging the oscilloscope readout with a rolling window matching the pulse length, followed by max-pooling with a window matching the laser repetition rate.

### Simulations

The metasurface spectra were simulated using the Frequency Domain Solver in CST Studio Suite with plane wave excitation. To calculate the back focal plane images, we performed sweeps of the inclination and azimuthal angles of the incident k-vector, with account of the symmetry of the structure.

Simulations of electromagnetic field distribution, thermal effects, and carrier dynamics were performed using COMSOL Multiphysics.

## Supplementary information

Supplementary Information

Description of Additional Supplementary Files

Supplementary Video 1

Transparent Peer Review file

## Data Availability

The data supporting the findings of this study are available within the Article and its Supplementary Information files.
