# Supplementary material for: Mid-IR light modulators enabled by dynamically tunable ultra-high-Q silicon membrane metasurfaces
Source: Nat Commun. 2026 Jul 17;17:8758. doi: 10.1038/s41467-026-75121-6 (PMC13493965; doi:10.1038/s41467-026-75121-6)
Supplement: Supplementary file 1 [file 41467_2026_75121_MOESM1_ESM.pdf]

Mid-IR Light Modulators Enabled by Dynamically Tunable Ultra High-Q Silicon Membrane Metasurfaces

– SUPPLEMENTARY INFORMATION –

Felix Ulrich Brikh,[†] Aleksei Ezerskii,[‡,¶] Olesia Pashina,[‡,§] Nikita Glebov,[†] Wenhong Yang,[†] Wenping Yin,[¶] Sergey V. Makarov,[*,¶,‡] Mihail Petrov,[‡] Ivan Sinev,[†] and Hatice Altug[*,†]

†*Institute of Bioengineering, École Polytechnique Fédérale de Lausanne (EPFL), Lausanne 1015, Switzerland*

‡*School of Physics and Engineering, ITMO University, St. Petersburg 197101, Russia*

¶*Qingdao Innovation and Development Center, Harbin Engineering University, Qingdao 266000, Shandong, China*

§*University of Brescia, Brescia, Italy*

E-mail: makarovsv@hrbeu.edu.cn; hatice.altug@epfl.ch

Contents

1 Estimation of thermo-optic coefficient 2

2 Electric analysis of doping 2

3 Optical analysis of doping **5**

4 Electro-thermal modulation **6**

5 Analysis of membrane cooling **7**

6 Charge carrier dynamics **8**

7 Comparison of High-Q Mid-IR Metasurfaces **14**

Supplementary Note 1 Estimation of thermo-optic coefficient

The thermo-optic coefficient of Si at $\lambda = 5.5$ µm for $T = 300\,\mathrm{K}$ is reported to be $\frac{dn}{dT} = 1.7 \cdot 10^{-4}\,\mathrm{K}^{-1}$.[1] However, the exact value dependents on the specific specimen and slightly varies with impurity and doping level. Therefore, we used our high-Q resonance to estimate the thermo-optic coefficient of the material at hand. In order to obtain the coefficient, we used a ceramic ring heater (10 W Thorlabs HT10KR1) with integrated thermistor to heat our metasurface to a specific temperature. Supplementary Figure 1a shows the observed position of the resonance wavelength and its tuning with temperature. Further analysis showed an excellent fit to a linear model (Supplementary Figure 1b). Combing the extracted temperature dependence with the theoretical resonance shift upon refractive index change as extracted from numerical simulations, we determine an effective thermo-optic coefficient of around $2 \cdot 10^{-4}\,\mathrm{K}^{-1}$.

Supplementary Note 2 Electric analysis of doping

The electric properties of the 1 µm doped Si membrane and contacts thereof were analyzed with a transfer length method. A pattern consisting of seven pairs of contact pads spaced with exponentially increasing distance from 250 µm to 1461 µm was fabricated. Supplementary Figure 2a shows the IV-curves for each distance, collected with a probe station (MPI

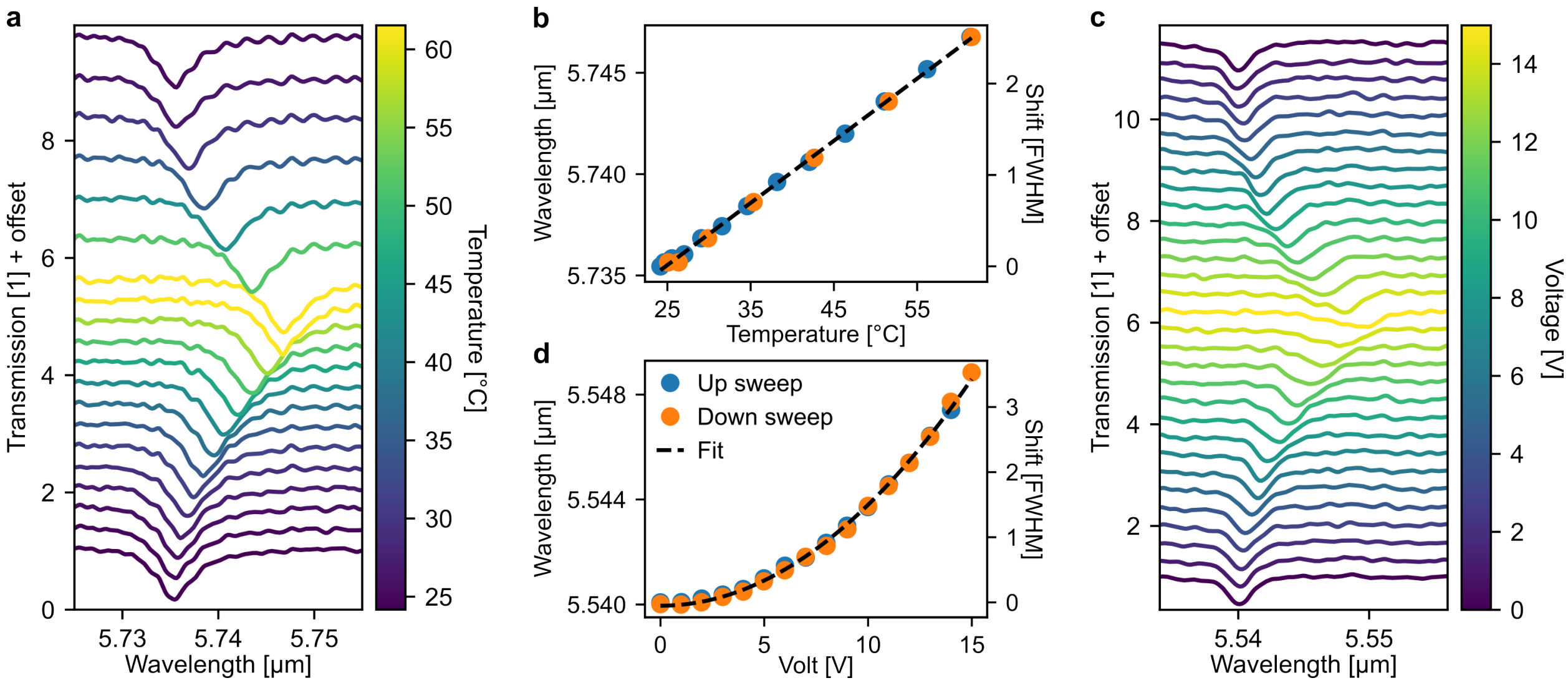

Supplementary Figure 1: **Static thermal and electro-thermal response**. (a,c) Spectra of a membrane metasurface for different temperatures and voltages, respectively, as indicated by color bar. Offset corresponds to measurement order. (b,d) Dependence of resonance wavelength as function of the corresponding physical stimuli. Coinciding up (blue) and down (orange) sweeps highlight excellence recovery.

TS150) connected to a semiconductor parameter analyzer (Agilent 4155B). The linear relation between applied voltage and measure current, confirms a ohmic contact. Hence, linear fitting of each curve allowed to extract the resistance for each distance, shown in Supplementary Figure 2b. The measured resistance is the sum of the membrane resistance and the two contact resistances at start and end or the membrane. Ignoring contact shape effects and assuming a conductive channel with constant width, the membrane resistance should grow linear with the distance. Hence, extrapolating the measured resistances to a infinite small contact separation yields the total contact resistance, which is twice the contact resistance R_c. From the geometric parameters of the contacts (700 µm × 250 µm) and membrane the cross section (700 µm × 1 µm) the specific contact resistivity ρ_c and specific membrane resistivity ρ_m can be calculated. The calculated values for four different doping levels are tabulated in Supplementary Table 1 .

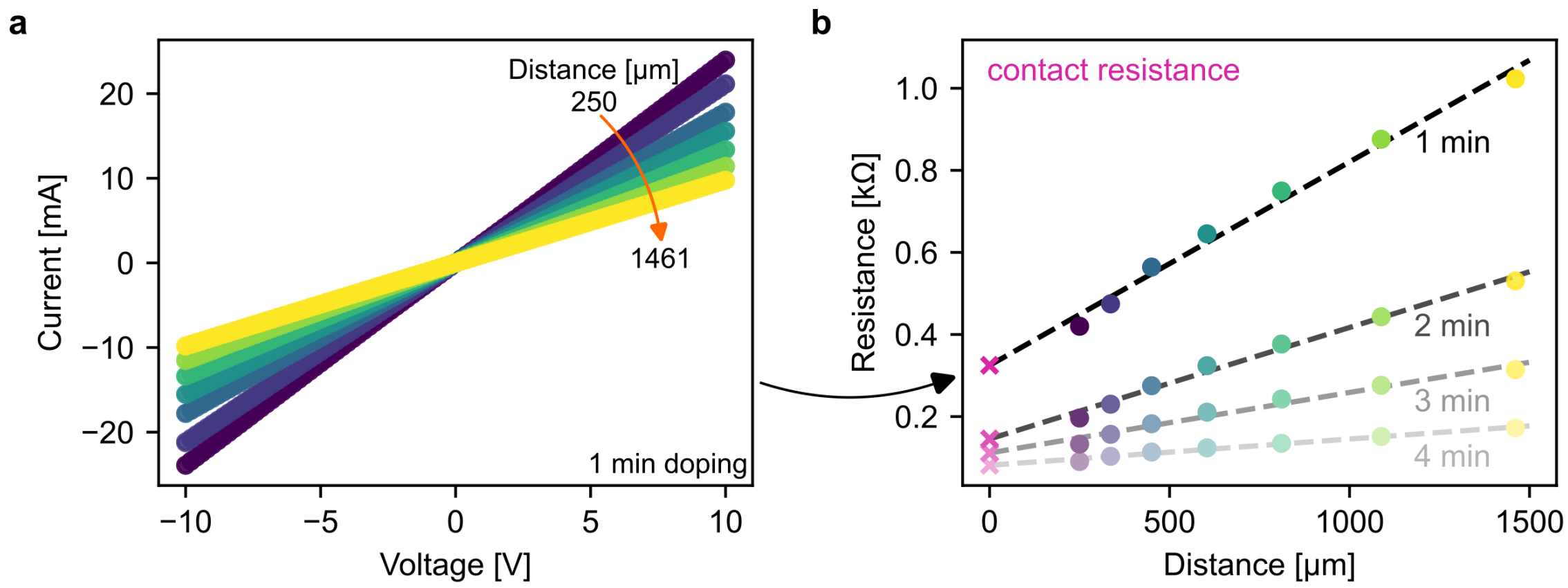

Supplementary Figure 2: **Electric properties of unstructured membrane**. (a) IV-curves of electricity flowing between two Ti-Al contact pads with separation between 250 μm to 1461 μm for a device silicon phosphorous doped for 1 min at 800 °C. (b) Separation of membrane (slope) and contact (y-intercept) resistance by transfer length method for doping levels between 1 min to 4 min.

Supplementary Table 1: Electric properties of the 1 μm thick Si membrane for various doping levels.

**Doping Time**	R_c (Ω)	R_m/L (Ω/cm)	ρ_c (Ω cm^2)	ρ_m (Ω cm)	$R_\square$ (Ω/□)
1 min	162.41	4958	0.284	$3.47 \cdot 10^{-2}$	347
2 min	72.71	2717	0.127	$1.90 \cdot 10^{-2}$	190
3 min	55.69	1473	0.098	$1.03 \cdot 10^{-2}$	103
4 min	40.66	640	0.071	$4.48 \cdot 10^{-3}$	44.8

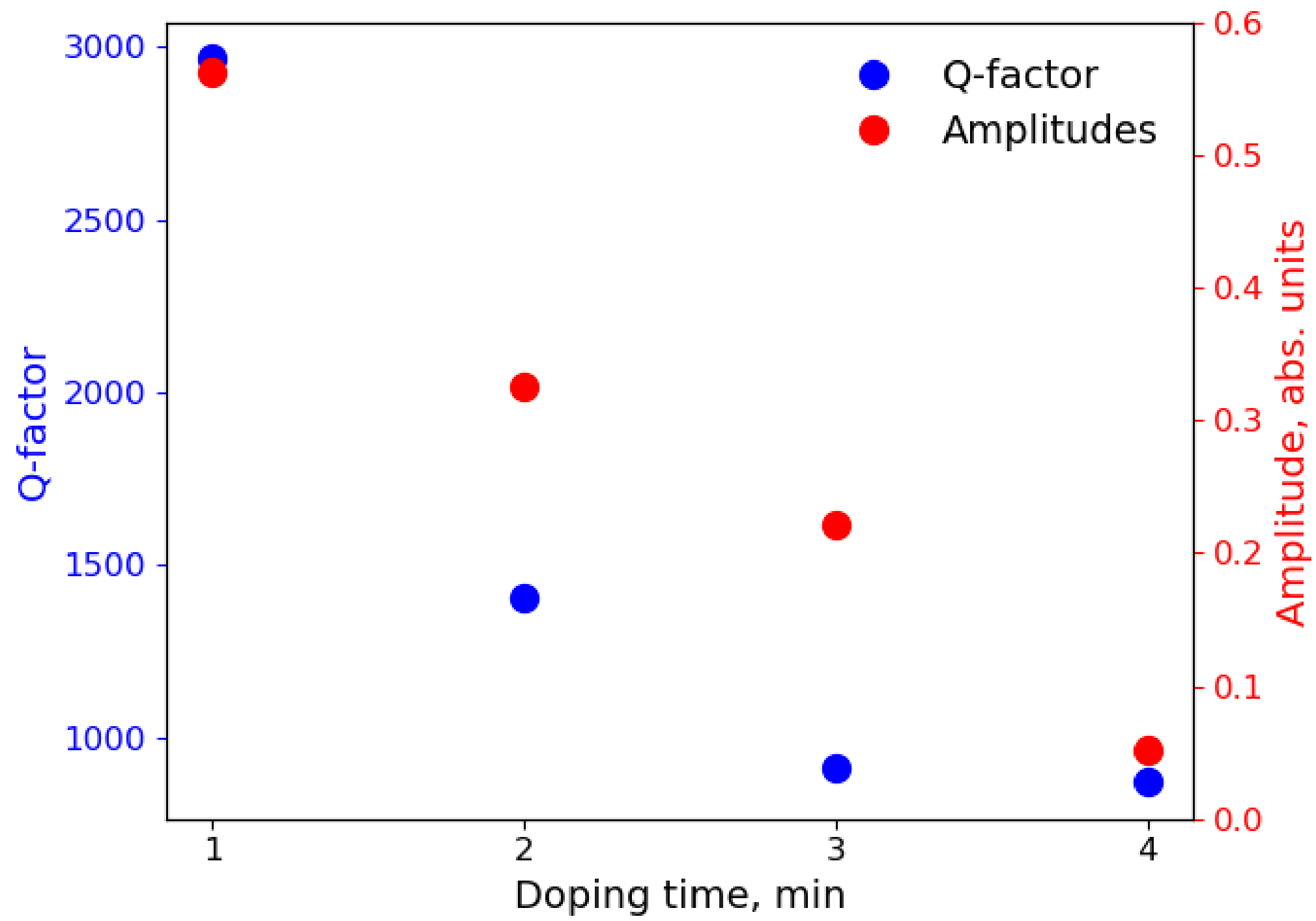

Supplementary Figure 3: **Optical characteristics of doped membranes** Q-factor (blue points) and resonance amplitude (red points) in dependence of doping time.

Supplementary Note 3 Optical analysis of doping

High doping levels contribute to increased optical losses as well as to changes in the real part of the refractive index. As a result, the Q-factor, resonance spectral position, and modulation amplitude can be affected. In practice, there is a trade-off between conductivity, and therefore membrane heating efficiency, and optical resonance quality.

Supplementary Figure 3 shows that with increasing doping time, both the Q-factor and resonance amplitude decrease, and by 4 minutes of doping, the resonances are strongly altered, exhibiting significantly reduced Q-factor and amplitude.

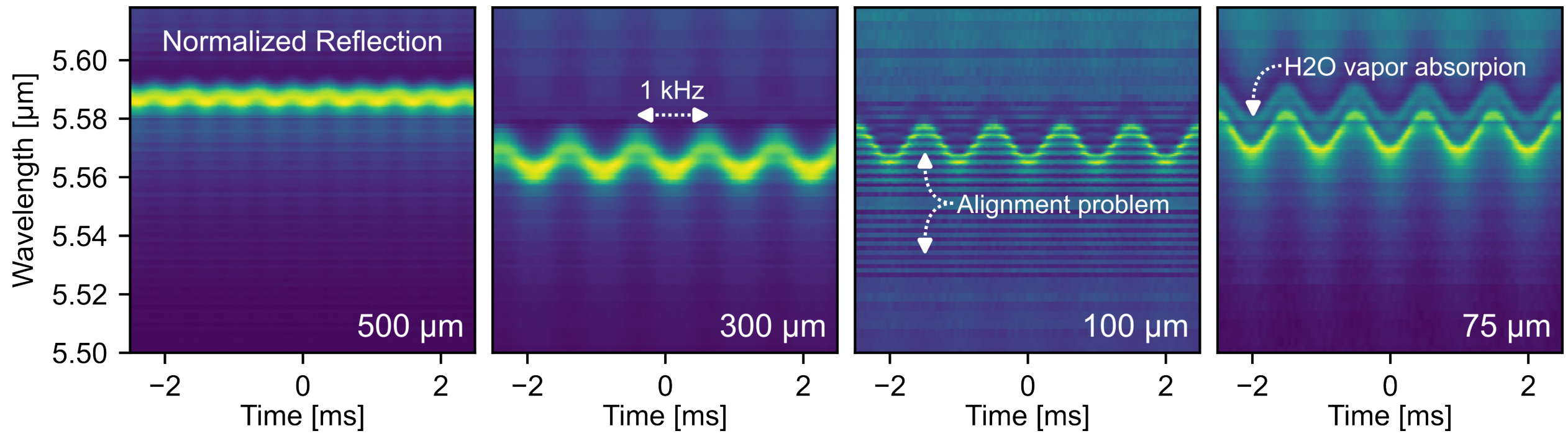

Supplementary Figure 4: **Time traces of electro-thermal modulation**. Time traces of normalized modulation at 1 kHz and 5 V driving voltage for metasurfaces with sizes from 500 µm to 75 µm. Annotation explain the major artifacts encountered.

Supplementary Note 4 Electro-thermal modulation

Supplementary Figure 4 shows the time traces recorded on the oscilloscope for different incident wavelengths at a modulation frequency of 1 kHz and driving voltage of 5 V. Each plot corresponds to a different membrane size. The plots reveal a stronger modulation for smaller membranes.

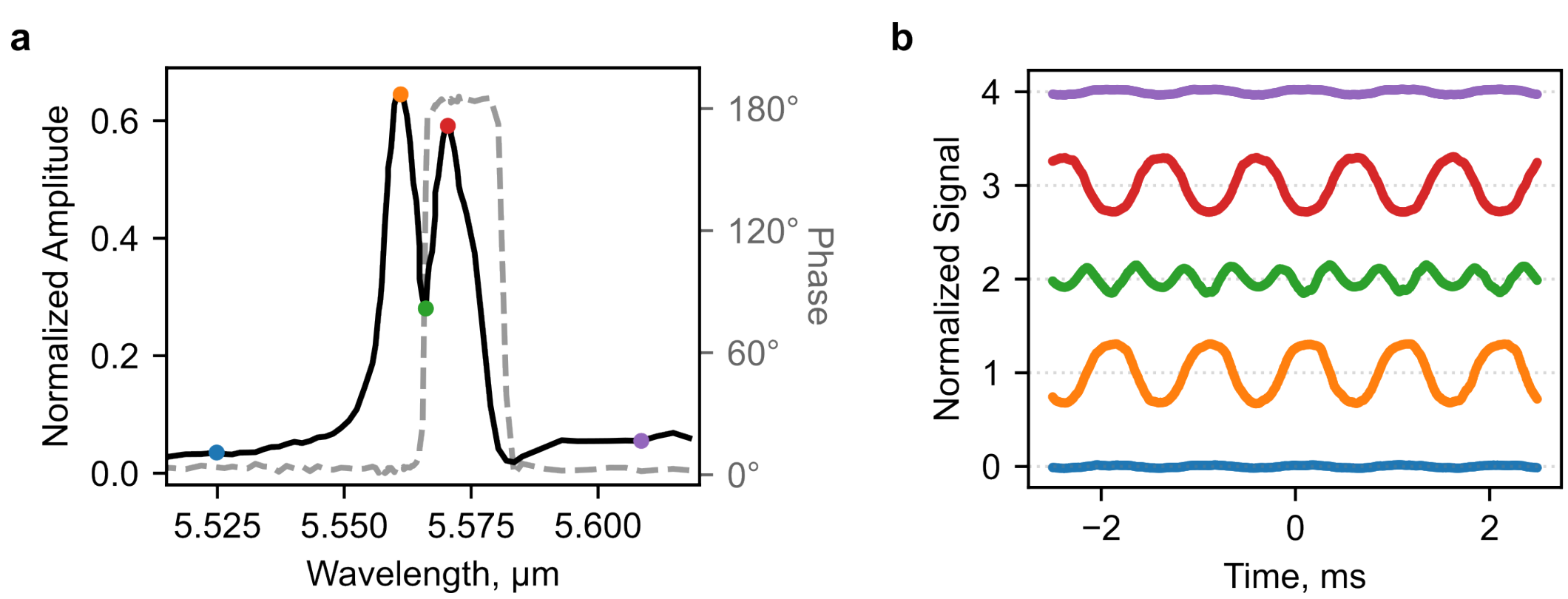

Supplementary Figure 5: **Modulation strength** a) Modulation strength (black, solid) and phase (gray, dashed) in dependence of probing wavelength. b) Time traces of single wavelength – indicated in panel a – recoded with oscilloscope, centered around zero and offset for convenience.

Supplementary Figure 5a shows the dependence of the modulation strength and oscillation phase with respect to the probing wavelength. Time traces at interesting points (*left off-resonance*, *original resonance*, *mid shift*, *maximally shifted*, *right off-resonance*) are

detailed in b). The careful reader might notice the 180° modulation phase shift between the *original* and *maximal shift* curves. The *mid shift* curve exhibits twice the modulation frequency as the optical resonance passes twice per power cycle.

Supplementary Note 5 Analysis of membrane cooling

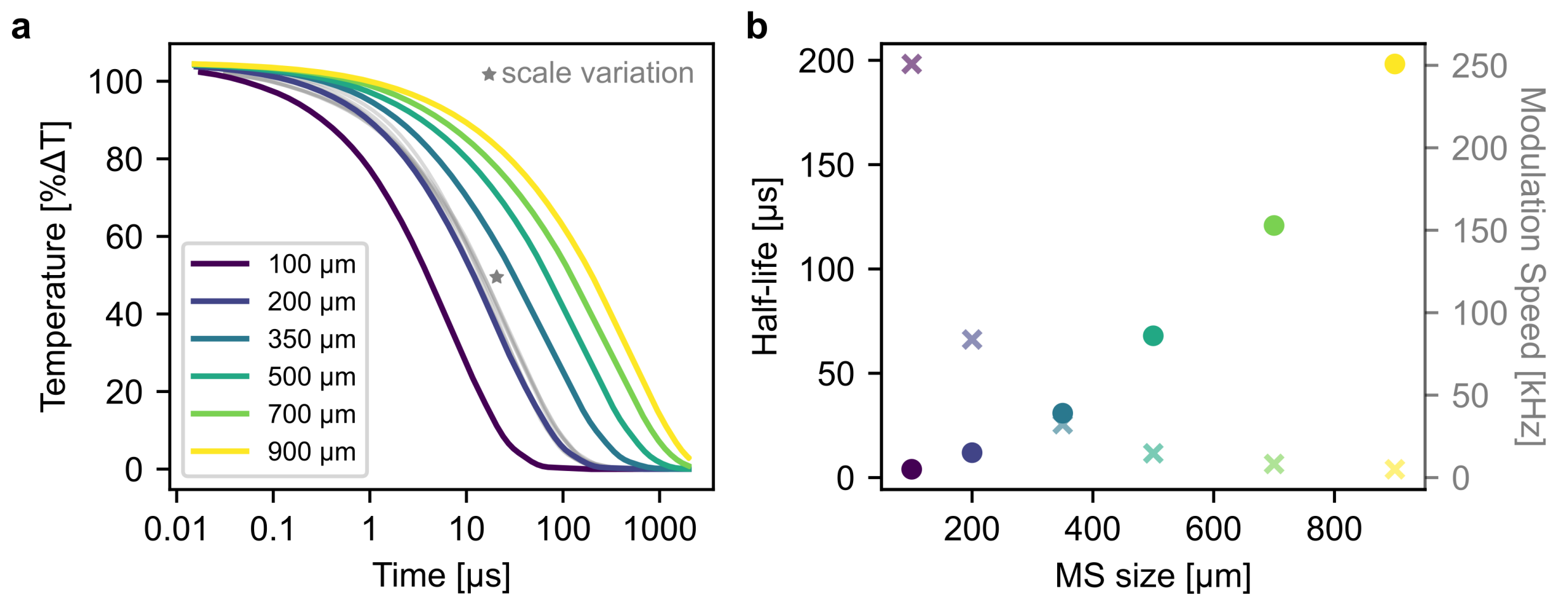

Supplementary Figure 6: **Membrane cooling**. (a) Average membrane temperature in percent of ΔT for square metasurfaces between 100 µm and 900 µm. Grey curves show the variation for different scaling factors of the elliptical openings. (b) Half-life of heat in membrane in dependence of metasurfaces size (circles, left axis) and corresponding maximum modulation speed (crosses, right axis).

We investigated the cooling behavior of the membrane of different sizes by heat simulations in COMSOL Multiphysics. At time $t = 0$ we set the membrane to temperature $T_0 = T_{\text{room}} + \Delta T$ and simulate its cooling over time. One challenge in the simulation was the different scales. On one hand we have a lateral dimension (membrane + electrodes) of several 100 µm, on the other hand we have thin layers of SiO_2 and Si as well as small elliptical openings $\sim$ 1 µm. For efficient simulations we scaled the ellipse opening by a factor of 5, drastically reducing the required computational resources. We verified the validity of this approach by sweeping the the scaling factor from 20 to 1 (real scale) for the 200 µm membrane, which resulted in minor deviation only (see grey curves in Supplementary Figure 6a).

The average heat of the membrane in dependence of time for different sized metasurfaces is shown in Supplementary Figure 6a). Smaller metasurfaces have a higher surface-to-volume ration, leading to faster cooling times. The evaluation of the half-life $\tau_{1/2}$ plotted in Supplementary Figure 6b confirms this statement. Assuming the membrane can be modulated maximally with $f_{\text{mod}} = \frac{1}{\tau_{1/2}}$, we plotted the expected modulation speed on the secondary y-axis. The resulting values are in good agreement with the measured data but the half-decay results in a reduced modulation depth.

Supplementary Note 6 Charge carrier dynamics

Extended two-temperature model. To quantify the optically induced carrier concentration in silicon membranes under femtosecond laser excitation, we employed a simplified model geometry — a homogeneous silicon film with the same thickness as the actual metasurface. This approximation is justified by the strong absorption regime at the pump wavelength (430 nm), where no high-quality metasurface modes are formed and the majority of the incident energy is absorbed in the subsurface region of the structure. As a result, the structural features of the metasurface have a negligible impact on carrier generation, validating the use of a continuous film as an effective substitute in the modeling.

The temporal evolution of the carrier concentration is modeled by accounting for carrier generation through optically induced interband transitions, followed by carrier thermalization, diffusion, relaxation, and energy exchange between the electronic and lattice subsystems. The modeling approach is based on the extended two-temperature model (eTTM)[2,3], which describes a system of coupled partial differential equations governing the spatiotemporal dynamics of the non-thermal electron energy density $\mathcal{U}_e^{\text{NT}}(\mathbf{r},t)$, the non-thermal $N_e^{\text{NT}}(\mathbf{r},t)$ and thermal $N_e^{\text{T}}(\mathbf{r},t)$ carrier concentrations, the electron $T_e(\mathbf{r},t)$ and phonon $T_{\text{ph}}(\mathbf{r},t)$ temperatures:

$$\begin{aligned}
\frac{\partial \mathcal{U}_e^{\mathrm{NT}}}{\partial t} &= \nabla \left[k_e \nabla \mathcal{U}_e^{\mathrm{NT}}\right] - (\Gamma_e + \Gamma_{\mathrm{ph}})\mathcal{U}_e^{\mathrm{NT}} + Q_{\mathrm{Ne}} \cdot \hbar\omega, \\
\frac{\partial N_e^{\mathrm{NT}}}{\partial t} &= Q_{\mathrm{Ne}} + \nabla \left[D(T_e)\nabla N_e^{\mathrm{NT}}\right] - \Gamma_e N_e^{\mathrm{NT}}, \\
\frac{\partial N_e^{\mathrm{T}}}{\partial t} &= \Gamma_e N_e^{\mathrm{NT}} + \nabla \left[D(T_e)\nabla N_e^{\mathrm{T}}\right] - \gamma_{\mathrm{rad}}(N_e^{\mathrm{T}})^2 \\
&\quad - \gamma_{\mathrm{Auger}}(N_e^{\mathrm{T}})^3 - \gamma_{\mathrm{surf}} N_e^{\mathrm{T}} \left[\delta(y - y_{\mathrm{surf1}}) + \delta(y - y_{\mathrm{surf2}})\right], \\
\frac{\partial (C_e T_e)}{\partial t} &= \nabla \left[k_e \nabla T_e\right] - G_{e\text{-}ph}(T_e - T_{ph}) + \Gamma_e \mathcal{U}_e^{\mathrm{NT}}, \\
\frac{\partial (C_{ph} T_{ph})}{\partial t} &= \nabla \left[k_{ph} \nabla T_{ph}\right] + G_{e\text{-}ph}(T_e - T_{ph}) + \Gamma_{\mathrm{ph}} \mathcal{U}_e^{\mathrm{NT}}.
\end{aligned}$$

Here $\mathcal{U}_e^{\mathrm{NT}}$ is energy density of non-thermalized electrons, N_e^{NT}, N_e^{T} are concentrations of non-thermalized and thermalized electrons, T_e, T_{ph} are temperatures of electrons and phonons. In addiation, C_e, C_{ph} are heat capacities of electrons and phonons for Si[4], k_e, k_{ph} are thermal conductivities[4], $D_a \approx \frac{2\mu_e\mu_h}{\mu_e+\mu_h}$ is ambipolar diffusion coefficient and μ_e, μ_{h} are electron and hole mobilities[5], Γ_e, Γ_{ph} are thermalization and electron–phonon coupling rates[5], γ_{rad}, γ_{Auger}, γ_{surf} are radiative[6], Auger [7] and surface[8] recombination rates, $\delta(y - y_{\mathrm{surf}})$ – delta-functions for the top and bottom surfaces of the Si film, $G_{e\text{-}ph}$ is electron–phonon coupling coefficient[4].

The carrier generation term $Q_{\mathrm{Ne}}(\mathbf{r}, t) = \frac{1}{2}\omega\varepsilon_0 \mathrm{Im}[\varepsilon(\mathbf{r})]|\mathbf{E}(\mathbf{r})|^2 \cdot \frac{\mathrm{GP}(t)}{\hbar\omega}$ describes the spatiotemporal profile of carriers excited by the pump with Gaussian pulse profile $\mathrm{GP}(t) = e^{-4\ln 2 t^2/\tau_p^2}$ with $\tau_p = 300\,\mathrm{fs}$ as the full width at half maximum of the pulse duration.

Simulations were performed using COMSOL Multiphysics, allowing for fully coupled modeling of electromagnetic field distribution, thermal effects, and carrier dynamics. Furthermore, we solved a self-consistent non-linear problem that accounts for the connection between light absorption and the dynamic modification of material properties. This modulation of optical properties under pump excitation arises from three principal mechanisms: Drude free-carrier response, band filling effect and bandgap renormalization[9]. Accurately capturing these effects is essential for describing the transient behavior of the dielectric func-

tion and the resulting carrier generation under intense ultrafast excitation, which can be further modified by self-action effects[10].

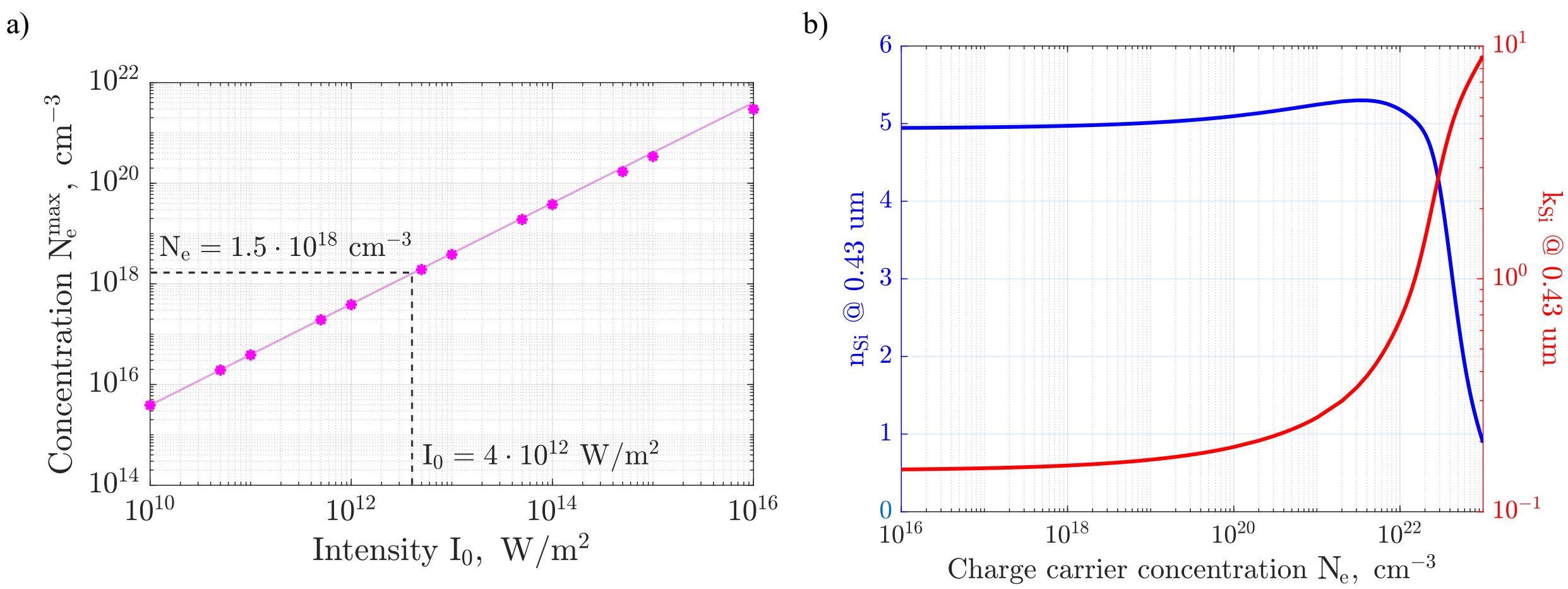

Supplementary Figure 7: (a) Peak charge carrier concentration $N_e^{\max}$ as a function of incident pump intensity I_0. The dashed lines mark the threshold intensity $I_0 = 4\times10^{12}$ W/m^2 required to reach charge carrier concentration $N_e = 1.5 \times 10^{18}$ cm^{-3}. (b) Calculated dependence of the real and imaginary part of the refractive index of crystalline silicon at 0.43 μm (pump wavelength) on the charge carrier density N_e.

We estimated the peak temporal values of the average carrier concentration N_e^{max} for different pump intensities I_0. Supplementary Figure 7 shows a clear linear dependence across different excitation levels, indicating the absence of self-action effects under the studied conditions. Indeed, to reach the self-action limit, we need a noticeable change in the complex refractive index upon excitation. However, at the pump wavelength, the combined effects of the Drude free-carrier response, band filling, and bandgap renormalization tend to partially cancel one another. As a result, a significant change in the complex refractive index only occurs at much higher carrier densities (Supplementary Figure 7b) that are unreachable under non-resonant excitation due to experimental intensity limits.

In contrast, under probe excitation, the optical response is governed primarily by the Drude mechanism, resulting in a more pronounced refractive index dependence on carrier density even at lower intensities. This underscores the advantage of probing in the mid-infrared spectral range compared to the visible, where compensating effects diminish the response. Additionally, high-Q resonances in the probe regime further enhance sensitiv-

ity, producing distinct spectral shifts from even small refractive index changes, making the system highly suitable for ultrafast carrier-induced optical modulation.

Finally, the model slightly underestimates the carrier concentration compared to experimental data, requiring approximately an order of magnitude higher pump intensity $I_0 = 4 \cdot 10^{12}$ W/m^2 to reach equivalent carrier densities $N_e = 1.5 \cdot 10^{18}$ cm^{-3}. This discrepancy is likely due to uncertainties in the effective focal spot size and differences in material parameters between the doped silicon used experimentally and the intrinsic silicon assumed in the model. Nevertheless, the model captures the key trends in ultrafast carrier dynamics and confirms the potential for achieving all-optical modulation in silicon membranes.

Analysis of Carrier Relaxation Time. We analyzed the relaxation dynamics of photoexcited carriers in silicon membranes by considering three principal recombination mechanisms: radiative, Auger, and surface recombination. The temporal evolution of the carrier density $N(t)$ after ultrafast excitation is described by the rate equation:

$$\frac{dN}{dt} = -\gamma_{\text{nrad}} N - \gamma_{\text{rad}} N^2 - \gamma_{\text{Auger}} N^3 - \gamma_{\text{surf}} N \left[\delta(y - y_{\text{surf1}}) + \delta(y - y_{\text{surf2}})\right], \qquad (1)$$

where γ_{nrad} – nonradiative (trap-assisted) recombination coefficient (assumed zero), $\gamma_{\text{rad}} = 1.1 \cdot 10^{-14}\,\text{cm}^3/\text{s}$ – radiative recombination coefficient[6], $\gamma_{\text{Auger}} = 3.8 \cdot 10^{-31}\,\text{cm}^6/\text{s}$ – Auger recombination coefficient[7], $\gamma_{\text{surf}} = 5 \cdot 10^4\,\text{cm/s}$ – surface recombination velocity (intermediate value from the range 10^2–10^5 cm/s)[8], $\delta(y - y_{\text{surf}})$ – delta-functions for the top and bottom surfaces of the Si membrane, N – photoexcited carrier concentration inside the structure. The total relaxation rate can be approximated by the combined contribution of bulk and surface processes:

$$\frac{1}{\tau_{\text{rel}}} = \Gamma_{\text{tot}} = \Gamma_{\text{bulk}} + \Gamma_{\text{surf}}, \qquad (2)$$

with bulk contribution $\Gamma_{\text{bulk}} = \gamma_{\text{rad}} N + \gamma_{\text{Auger}} N^2$, and surface contribution $\Gamma_{\text{surf}} = \frac{2\gamma_{\text{surf}}}{h}$, where $h = 1\,\mu\text{m} = 1 \cdot 10^{-4}\,\text{cm}$ is the membrane thickness. Substituting the maximum carrier

concentration $N_{\max} = 1.4 \cdot 10^{18}\,\mathrm{cm}^{-3}$, we obtain:

$$\gamma_{\mathrm{rad}} N = \left(1.1 \cdot 10^{-14}\,\mathrm{cm}^3/\mathrm{s}\right) \cdot \left(1.4 \cdot 10^{18}\,\mathrm{cm}^{-3}\right) = 1.54 \cdot 10^{4}\,\mathrm{s}^{-1},$$
$$\gamma_{\mathrm{Auger}} N^2 = \left(3.8 \cdot 10^{-31}\,\mathrm{cm}^6/\mathrm{s}\right) \cdot \left(1.4 \cdot 10^{18}\,\mathrm{cm}^{-3}\right)^2 = 7.45 \cdot 10^{5}\,\mathrm{s}^{-1},$$
$$\frac{\gamma_{\mathrm{surf}}}{h} = \frac{5 \cdot 10^{4}\,\mathrm{cm/s}}{1 \cdot 10^{-4}\,\mathrm{cm}} = 5 \cdot 10^{8}\,\mathrm{s}^{-1}.$$

This analysis demonstrates that, for the given maximum carrier concentration, surface recombination dominates the total recombination rate by several orders of magnitude compared to other mechanisms. Although at higher carrier concentrations – achievable by increasing the optical pumping power – Auger recombination may eventually become dominant, this is not the case in the present study. Accordingly, the effective total recombination rate can be approximated as:

$$\Gamma_{\mathrm{tot}} \approx \Gamma_{\mathrm{surf}} = 10^{9}\,\mathrm{s}^{-1}, \tag{3}$$

which corresponds to a relaxation time of:

$$\tau_{\mathrm{rel}} = \frac{1}{\Gamma_{\mathrm{tot}}} \approx 1\,\mathrm{ns}. \tag{4}$$

This value is in good agreement with the relaxation time mentioned in the main text (approximately 1–2 ns), confirming that in thin Si membranes, the carrier lifetime is primarily defined by surface recombination due to the high surface-to-volume ratio as well as additional surface roughness generated during the etching process.

Spectral dependence of probe refractive index modulation. The modulation of the complex refractive index due to the photoexcited free carriers in the mid-infrared spectral range was evaluated using the Drude model[9], which accounts for the free-carrier response and is the dominant mechanism for dielectric permittivity changes at wavelengths significantly longer than the bandgap edge. Under these conditions, band filling and band structure

modifications can be neglected, simplifying the analysis of the optical response.

The dispersion of the induced changes in refractive index (Δn) and extinction coefficient (Δk) was calculated for a carrier concentration of $N = 1.5 \cdot 10^{18}\,\mathrm{cm}^{-3}$, which corresponds to the maximum excitation level achieved in the studied silicon membrane structure. The total complex refractive index was computed as:

$$\tilde{n}(\omega) + i\tilde{k}(\omega) = n_0(\omega) + \Delta n(\omega) + i\left[k_0(\omega) + \Delta k(\omega)\right],$$

where n_0 and k_0 represent the unperturbed refractive index and extinction coefficient of silicon. The modulation components Δn and Δk were obtained from the real and imaginary parts of permittivity modulation based on Drude model[9]:

$$\Delta\varepsilon(\omega) = -\frac{Ne^2}{\varepsilon_0 m^* \omega^2 + i\omega\gamma},$$

where $m^* = 0.18\ m_e$ - the effective carrier mass, $\gamma = 5 \times 10^{14}\ \mathrm{s}^{-1}$ - the carrier scattering rate, $N = 1.4 \cdot 10^{18}\,\mathrm{cm}^{-3}$ - the carrier concentration.

The resulting spectral curves of Δn and Δk (Supplementary Figure 8) demonstrate notable dispersion in the vicinity of the probe wavelength. At $\lambda = 5650$ nm, the real part change is approximately $\Delta n = \tilde{n} - n_0 \approx -0.034$, indicating a sufficient decrease in refractive index to produce a measurable blueshift in optical resonances. Importantly, while the real part exhibits a meaningful modulation, the imaginary part $\Delta k = \tilde{k} - k_0$ remains relatively small. This ensures that the resonance shift is observable without significant broadening or attenuation of the mode. Thus, the chosen probe wavelength offers a favorable balance between index sensitivity and absorption losses, which is advantageous for photonic modulation or sensing applications in the mid-IR regime.

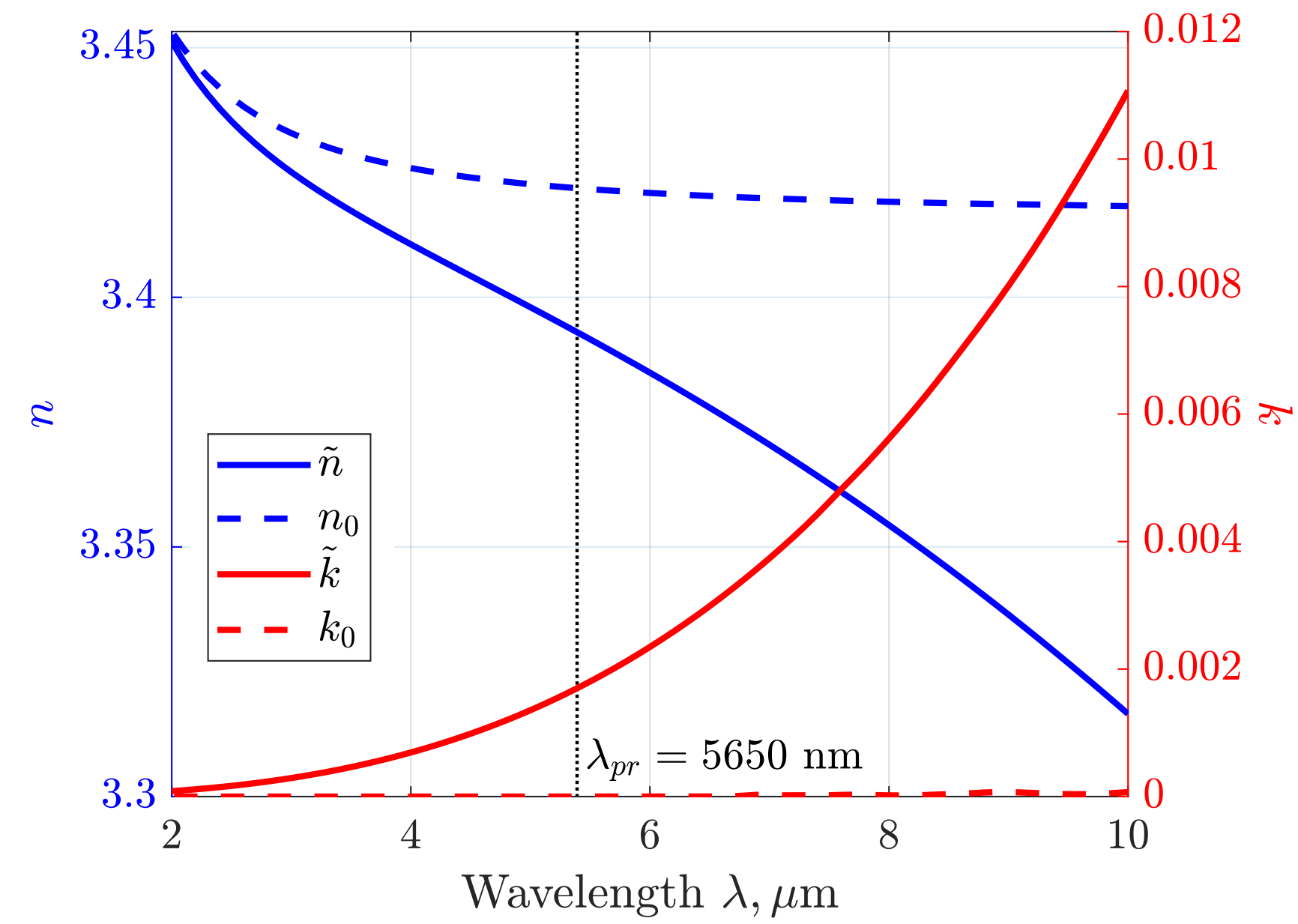

Supplementary Figure 8: Spectral dependence of the refractive index n (blue lines) and extinction coefficient k (red lines) calculated using the Drude model for a carrier density of $N_e^{\max} = 1.5 \times 10^{18}$ cm^{-3}. Solid lines correspond to the unperturbed refractive index $n_0 + ik_0$ and extinction coefficient, while dashed lines show their modulated value $\tilde{n} + i\tilde{k}$ due to photoexcited free carriers. The vertical dotted line indicates the probe wavelength $\lambda = 5650$ nm.

Supplementary Note 7 Comparison of High-Q Mid-IR Metasurfaces

Higher Q-factors correspond to narrower resonances and often desired stronger light-matter interactions. However, very high-Q designs often exhibit reduced resonance amplitudes due to mismatch in radiative and non-radiative losses. Supplementary Table 2 summarizes experimentally realized high-Q mid-IR metasurfaces, comparing their quality factors and resonance amplitudes. The comparison focuses on the spectral range from 4 µm to 10 µm, which covers the important mid-IR molecular fingerprint region. The table includes both plasmonic and dielectric metasurfaces, as well as recent silicon membrane designs. In addition, topological modes in silicon on sapphire substrates with Q-factors around 5000 have been reported[11], but their amplitudes are expected to be very low and sapphire limits the range

to <6 µm.

Supplementary Table 2: Q-factors and resonance amplitudes of mid-IR metasurfaces (4–10 µm). Sorted by increasing Q-factor.

Material Stack	Q factor	Amplitude	Reference
Ge / CaF_2	75	0.7	Jangid, 2024[12]
Au / Si	92	0.8	Nishijima, 2022[13]
Si / SiO_2 / Si	94	0.2	Wu, 2014[14]
Ge / CaF_2	100	0.65	Richter, 2024[15]
Si / CaF_2	115	0.5-1	Tittl, 2018[16]
ITO / Au	200	0.8-1	Li, 2014[17]
Si membrane	722	0.7	Adi, 2024[18]
Si membrane	734	0.3	Rosas, 2025[19]
**Si membrane**	**2965**	**0.55**	**this work**

References

1. Frey, B. J.; Leviton, D. B.; Madison, T. J. Temperature-dependent refractive index of silicon and germanium. Optomechanical Technologies for Astronomy. 2006.

2. Sivan, Y.; Spector, M. Ultrafast dynamics of optically induced heat gratings in metals. *ACS Photonics* **2020**, *7*, 1271–1279.

3. Pashina, O.; Sergaeva, O.; Seredin, A.; De Angelis, C.; Petrov, M. Ultrafast excitation of surface plasmon-polariton through optically induced diffraction grating in GaAs films. 2024 Eighteenth International Congress on Artificial Materials for Novel Wave Phenomena (Metamaterials). 2024; pp 1–3.

4. Derrien, T. J.-Y.; Bulgakova, N. M. Modeling of silicon in femtosecond laser-induced modification regimes: Accounting for ambipolar diffusion. Nonlinear Optics and Applications X. 2017; pp 72–85.

5. Bulgakova, N.; Stoian, R.; Rosenfeld, A.; Hertel, I.; Campbell, E. Electronic transport

and consequences for material removal in ultrafast pulsed laser ablation of materials. *Physical Review B* **2004**, *69*, 054102.

6. Schlangenotto, H.; Maeder, H.; Gerlach, W. Temperature dependence of the radiative recombination coefficient in silicon. *physica status solidi (a)* **1974**, *21*, 357–367.

7. Chen, J.; Tzou, D.; Beraun, J. Numerical investigation of ultrashort laser damage in semiconductors. *International Journal of Heat and Mass Transfer* **2005**, *48*, 501–509.

8. Mackel, H.; Cuevas, A. Determination of the surface recombination velocity of unpassivated silicon from spectral photoconductance measurements. 3rd World Conference onPhotovoltaic Energy Conversion, 2003. Proceedings of. 2003; pp 71–74.

9. Sokolowski-Tinten, K.; Von der Linde, D. Generation of dense electron-hole plasmas in silicon. *Physical Review B* **2000**, *61*, 2643.

10. Sinev, I. S.; Koshelev, K.; Liu, Z.; Rudenko, A.; Ladutenko, K.; Shcherbakov, A.; Sadrieva, Z.; Baranov, M.; Itina, T.; Liu, J.; others Observation of ultrafast self-action effects in quasi-BIC resonant metasurfaces. *Nano Letters* **2021**, *21*, 8848–8855.

11. Chen, K.; Komissarenko, F.; Smirnova, D.; Vakulenko, A.; Kiriushechkina, S.; Volkovskaya, I.; Guddala, S.; Menon, V.; Alù, A.; Khanikaev, A. B. Photonic Dirac cavities with spatially varying mass term. *Science Advances* **2023**, *9*.

12. Jangid, P.; Richter, F. U.; Tseng, M. L.; Sinev, I.; Kruk, S.; Altug, H.; Kivshar, Y. Spectral Tuning of High-Harmonic Generation with Resonance-Gradient Metasurfaces. *Advanced Materials* **2023**, *36*.

13. Nishijima, Y.; Morimoto, S.; Balčytis, A.; Hashizume, T.; Matsubara, R.; Kubono, A.; To, N.; Ryu, M.; Morikawa, J.; Juodkazis, S. Coupling of molecular vibration and metasurface modes for efficient mid-infrared emission. *J. Mater. Chem. C* **2022**, *10*, 451–462.

14. Wu, C.; Arju, N.; Kelp, G.; Fan, J. A.; Dominguez, J.; Gonzales, E.; Tutuc, E.; Brener, I.; Shvets, G. Spectrally selective chiral silicon metasurfaces based on infrared Fano resonances. *Nature Communications* **2014**, *5*.

15. Richter, F. U.; Sinev, I.; Zhou, S.; Leitis, A.; Oh, S.-H.; Tseng, M. L.; Kivshar, Y.; Altug, H. Gradient High-Q Dielectric Metasurfaces for Broadband Sensing and Control of Vibrational Light-Matter Coupling. *Adv. Mater.* **2024**, *36*, 2314279.

16. Tittl, A.; Leitis, A.; Liu, M.; Yesilkoy, F.; Choi, D.-Y.; Neshev, D. N.; Kivshar, Y. S.; Altug, H. Imaging-based molecular barcoding with pixelated dielectric metasurfaces. *Science* **2018**, *360*, 1105–1109.

17. Li, S.-Q.; Zhou, W.; Bruce Buchholz, D.; Ketterson, J. B.; Ocola, L. E.; Sakoda, K.; Chang, R. P. H. Ultra-sharp plasmonic resonances from monopole optical nanoantenna phased arrays. *Applied Physics Letters* **2014**, *104*.

18. Adi, W.; Rosas, S.; Beisenova, A.; Biswas, S. K.; Mei, H.; Czaplewski, D. A.; Yesilkoy, F. Trapping light in air with membrane metasurfaces for vibrational strong coupling. *Nature Communications* **2024**, *15*.

19. Rosas, S.; Adi, W.; Beisenova, A.; Biswas, S. K.; Kuruoglu, F.; Mei, H.; Kats, M. A.; Czaplewski, D. A.; Kivshar, Y. S.; Yesilkoy, F. Enhanced biochemical sensing with high-Q transmission resonances in free-standing membrane metasurfaces. *Optica* **2025**, *12*, 178.
