# Supplementary material for: Mid-IR light modulators enabled by dynamically tunable ultra-high-Q silicon membrane metasurfaces
Source: Nat Commun. 2026 Jul 17;17:8758. doi: 10.1038/s41467-026-75121-6 (PMC13493965; doi:10.1038/s41467-026-75121-6)
Supplement: Supplementary file 2 [file 41467_2026_75121_MOESM2_ESM.pdf]

Description of Additional Supplementary Files

Supplementary Video 1: Spectral dependence of the back focal plane images of silicon membrane metasurface (left) and its comparison to the simulation (right).
