# Supplementary material for: Mid-IR light modulators enabled by dynamically tunable ultra-high-Q silicon membrane metasurfaces
Source: Nat Commun. 2026 Jul 17;17:8758. doi: 10.1038/s41467-026-75121-6 (PMC13493965; doi:10.1038/s41467-026-75121-6)
Supplement: Supplementary file 3 [file 41467_2026_75121_MOESM4_ESM.pdf]

nature portfolio

Peer Review File

Mid-IR Light Modulators Enabled by Dynamically Tunable Ultra High-Q Silicon Membrane Metasurfaces

Corresponding Author: Dr Ivan Sinev

This file contains all reviewer reports in order by version, followed by all author rebuttals in order by version.

Version 0:

Reviewer comments:

Reviewer #1

(Remarks to the Author)
The manuscript reports a free-space mid-infrared silicon membrane metasurface with electrically and optically tunable resonant response. While the device is carefully fabricated and experimentally characterized, the work primarily represents an incremental engineering refinement of previously reported structures and tuning mechanisms. Moreover, the physical description of the tuning processes lacks conceptual precision, further weakening the manuscript's claims of novelty and significance. Overall, the work does not meet the standards required for publication in Nature Communications.
Major Concerns
1. The central device platform—a free-standing (or membrane-type) silicon metasurface operating under free-space mid-infrared excitation—has already been established in prior literature. In particular, free-standing silicon membrane metasurfaces supporting high-Q resonances (including qBIC-type modes) in the mid-infrared have been demonstrated and analyzed in detail in recent high-impact publications (e.g., Nature Communications 15, 1–9 (2024), DOI: 10.1038/s41467-024-54284-0).

In this context, the structural design presented here does not constitute a new class of photonic system, but rather a variation within an already well-explored design space.
2. The manuscript emphasizes both electrical and optical tuning. However, both tuning routes rely on well-known material responses of silicon:

Electrical tuning proceeds via electro-thermal heating followed by the thermo-optic effect.

Optical tuning is mediated by optically generated charge carriers, which modify the refractive index and absorption through free-carrier dispersion and absorption, often accompanied by secondary heating.

Thermo-optic tuning of semiconductor metasurface resonators, including silicon, has been systematically studied and reviewed, with detailed discussions of resonance shifts, linewidth evolution, and quality-factor dependence on temperature (see Lewi et al., Nanophotonics 8, 331–338 (2019), DOI: 10.1515/nanoph-2018-0178).

Similarly, photoinduced modulation in silicon metasurfaces—where optically generated carriers lead to simultaneous spectral shift and Q variation—has been explicitly reported in recent literature (e.g., Tognazzi et al., Advanced Photonics 5, 066006 (2023), DOI: 10.1117/1.AP.5.6.066006).
3. The manuscript repeatedly employs the term electro-optic to describe the electrically driven tuning process. Conventionally, electro-optic effects refer to field-driven refractive-index modulation (e.g., Pockels or Kerr effects).

In the present work, the electrical control modifies the optical response indirectly through heating and carrier-related processes, rather than through a direct electric-field–induced refractive-index change. Referring to this mechanism as "electro-optic" therefore conflates actuation pathways with optical modulation mechanisms and introduces conceptual ambiguity.
4. Given that both the device architecture and tuning mechanisms have clear precedents, the primary contribution of the manuscript lies in engineering refinement, such as geometry optimization or resonance shaping.
While such refinements are valuable from an implementation standpoint, they do not establish a new physical regime, scaling law, or modulation paradigm.

5. Although relatively high quality factors are reported, the tuning process—whether electrical or optical—is accompanied by continuous spectral shifting of the resonance, during which both the resonance wavelength and the Q factor evolve.

As a result, the high-Q resonance does not represent a stable operating point during modulation. The observed intensity variation largely arises from the resonance moving across a fixed probe wavelength, limiting the functional benefit of achieving a high Q factor in this configuration.
6. Even when evaluated as an engineering implementation, the reported performance does not clearly surpass existing approaches. For example, thermo-optically tunable mid-infrared silicon metasurface filters have already demonstrated controlled, reversible spectral tuning with well-defined application contexts (Russell et al., Laser & Photonics Reviews (2025), DOI: 10.1002/lpor.202400853).

By comparison, the present manuscript does not demonstrate superior modulation depth, speed, energy efficiency, or stability. Intrinsic limitations associated with thermal diffusion and carrier dynamics further constrain achievable modulation speed and energy consumption, and no evidence is provided that these limits are meaningfully improved.

Reviewer #2

(Remarks to the Author)
This manuscript presents a suspended silicon membrane platform for the mid-infrared (5–6 µm) that utilizes qBIC resonances to achieve high Q-factors (~2500). The authors demonstrate two modulation schemes: electro-thermal tuning and ultrafast all-optical modulation. This work aims to overcome substrate loss limitations to create a scalable, CMOS-compatible modulator. The suspended silicon platform is technically sound, the reported Q-factors are impressive, and the dynamic modulation is considered effective in this field. The manuscript is logically organized, and the conclusions are supported by the presented results. The manuscript can be further strengthened by addressing the following comments.

1. The manuscript claims "record-high measured Q-factors up to 3000" in the abstract. However, it is just one data point in Figure 2e. The transmission spectrum corresponding to the highest reported Q-factor should be provided. The claim is also inconsistent in the manuscript. In Table 2 in the Supporting Information, the authors use 2500 for this work to be compared with others.
2. The authors use phosphorous doping to improve heating efficiency. However, there is no comparison data demonstrating how the doping affects q-factors, i.e., the same device before and after doping.
3. The manuscript claims wafer-scale fabrication compatible with the demonstration of direct laser writing. However, there is only one datapoint for the DLW sample. It is not clear if the SEM image of Figure 2c is from DLW or EBL. Also, no consistency analysis, design/device mismatch, or imperfection analysis is performed for DLW samples. This is critical for feature sizes close to equipment limitations.
4. Line 119, "we keep the hole area constant at A=1.1um2". However, Figure 2c indicates that the hole radius is larger than 1 µm. Wrong scale bar?
5. It is not clear how the infrared transmission spectra in Figures 5d and 5h are obtained. As far as the reviewer knows, the KYMERA-193i-B2 spectrograph is for Vis-NIR. A detailed description of the MIR measurement is helpful for the reader.

Reviewer #3

(Remarks to the Author)
This paper reports a mid-infrared optical light modulator based on high-Q silicon metasurfaces. The authors demonstrate thermally and optically tunable transmission resonances around a wavelength of 5 µm. Very high quality factors are achieved using bound-state-in-the-continuum (BIC)–type resonances.

The paper is well written, and the results are sound and interesting. I recommend this paper for publication in Nature Communications, subject to minor revisions.

I have a few minor comments that may help to improve the manuscript:

1. The physical origin of the BIC resonance is not discussed in sufficient detail. A clearer explanation of the nature of this resonance and the mechanism leading to the high-Q response would strengthen the paper.

2. The experimental method used to measure the BIC resonance at normal incidence is not sufficiently clear. In particular, the authors should explain why a spatial filter is required and discuss whether the filter could introduce any artificial resonance effects.

3.It would be beneficial to compare this approach with other infrared-active materials and modulation platforms, such as carbon nanotubes and graphene, to better contextualize the advantages and limitations of the proposed system.

4. the potential applications of this method should be discussed more concretely. The authors mention possible use in quantum photonics, but this claim is not sufficiently supported and would benefit from clarification or justification.

Reviewer #4

(Remarks to the Author)

I co-reviewed this manuscript with one of the reviewers who provided the listed reports. This is part of the Nature Communications initiative to facilitate training in peer review and to provide appropriate recognition for Early Career Researchers who co-review manuscripts.

Version 1:

Reviewer comments:

Reviewer #1

(Remarks to the Author)
I thank the authors for their detailed rebuttal and the substantial experimental efforts made during the revision. The implementation of the wafer-scale DUV lithography protocol and the supplementary doping-time optimization data are well received. The technical execution of achieving a measured Q-factor approaching 3000 in a free-standing silicon membrane is highly acknowledged.

However, while the fabrication quality is solid, the manuscript fundamentally remains an incremental technical refinement. Moving an established device architecture to the mid-infrared (mid-IR) spectrum is a valuable engineering application, but it cannot serve as a justification for limited device-level performance and a lack of fundamental physical novelty. Several critical bottlenecks regarding performance and practical viability remain unaddressed:

1. Architectural Novelty and Mechanism Translation
The authors heavily defend their novelty by claiming this is the "first use of high-Q silicon membranes for fast free-space mid-IR light modulation," attempting to distinguish their work from prior static SEIRA sensors. However, the fundamental paradigm of utilizing asymmetric, free-standing silicon nanostructures (breaking symmetry to excite high-Q modes) for active free-space spatial light modulation is already well-established. For instance, Kwon et al. (Nat. Commun. 13, 5811, 2022, DOI: 10.1038/s41467-022-33449-9) and Zheng et al. (Nano Lett. 23, 5588-5594, 2023, DOI: 10.1021/acs.nanolett.3c00999) have already demonstrated highly efficient amplitude and phase modulation using perturbed suspended silicon membranes without bottom mirrors.

Transitioning this exact structural paradigm from the near-infrared to the mid-infrared spectrum represents a solid engineering improvement through wavelength adaptation, rather than a fundamental breakthrough. In fact, scaling the operating wavelength into the mid-IR inherently relaxes the fabrication constraints. Because the critical feature sizes of the meta-atoms scale up proportionally with the longer wavelength, the lithographic resolution requirements are lowered, making the device fabrication physically easier and more forgiving. Coupling this up-scaled static structural paradigm with the classical tuning mechanisms of silicon (thermo-optic and free-carrier dispersion) does not establish a fundamentally new light-matter interaction regime warranting publication in a general-interest, high-impact journal.

2. The Overstatement of Mid-IR Scarcity and Speed Bottlenecks
The authors highlight a supposed scarcity of actively tunable mid-IR devices to justify their metrics. However, this assertion misrepresents the current state-of-the-art, which has already advanced to highly integrated, 2D-addressable architectures with superior speeds.

For instance, Popescu et al. (arXiv:2511.03583, 2025) have recently demonstrated a fully 2D-addressable mid-IR transmissive spatial light modulator utilizing CMOS-foundry microheaters and integrated PIN diode selectors. In comparison, the bulk interdigitated heating architecture presented in this manuscript is architecturally primitive and lacks true spatial addressability. Furthermore, regarding the authors' claim of "fast" electro-thermal tuning (14.5 kHz), recent works have vastly outperformed this metric. Liu et al. (Nat. Commun. 16:1665, 2025, DOI: 10.1038/s41467-025-56811-z) recently demonstrated electrically programmable mid-IR metasurfaces utilizing quasi-bound states in the continuum (QBIC)—the exact resonance physics claimed here—achieving electro-thermal modulation speeds of ~146 kHz. In light of these concurrent mid-IR advancements, the present manuscript's electro-thermal speed (14.5 kHz) represents an evolutionary technical upgrade within expected thermal constraints, rather than a forward-looking paradigm shift.

3. Physical Instability and Artificial Testing Environment
With a measured Q-factor near 3000, the resonance linewidth is exceptionally narrow. The authors state that this sharp spectral slope is utilized to enhance modulation depth with low driving amplitude. However, the current demonstration is highly idealized and fragile.

To even capture this resonance, a custom FTIR accessory with extreme spatial filtering was required to choke the effective NA down to 0.02. In any real-world free-space mid-IR deployment (e.g., coupling with narrowband quantum cascade lasers or thermal emission management), light beams carry inherent angular divergence. Furthermore, even minor environmental thermal fluctuations or laser source frequency jitters will cause the bias point to drift completely off this narrow slope. Without demonstrating long-term operational stability or a robust feedback-locking mechanism, the functional benefit of this high-Q configuration in real-world applications remains unsubstantiated.

4. The Illusion of System-Level Low Power Consumption
For the all-optical modulation route, the authors highlight that the required pump fluence is two orders of magnitude lower than that in the near-infrared or visible ranges. However, from a practical, system-level perspective, this scheme inherently requires an external, bulky, and power-hungry ultra-fast pulsed laser system to act as the pump control. Minimizing the

energy absorption on the metasurface chip at the expense of introducing a high-overhead external optical setup does not translate to genuine "low-power integrated photonics." The overall system footprint severely undermines the claimed practical integration advantages.

Summary:
In conclusion, this work represents a piece of good engineering work with high-quality fabrication optimization. However, because the fundamental physics are a wavelength-scaled translation of known paradigms, the operating speeds are thermally constrained and outpaced by recent literature, and the demonstration remains a highly artificial laboratory setup, the manuscript in its current form does not exhibit the transformative breakthroughs expected for Nature Communications.

Reviewer #2

(Remarks to the Author)
All my previous comments have been satisfactorily addressed. The only issue is that “HARPIA-TA” is not added in the revised manuscript.

Reviewer #3

(Remarks to the Author)
The revised manuscript addresses all my points. I recommend it for publication in Nature Communications.

Reviewer #4

(Remarks to the Author)
I co-reviewed this manuscript with one of the reviewers who provided the listed reports. This is part of the Nature Communications initiative to facilitate training in peer review and to provide appropriate recognition for Early Career Researchers who co-review manuscripts.

Open Access This Peer Review File is licensed under a Creative Commons Attribution 4.0 International License, which permits use, sharing, adaptation, distribution and reproduction in any medium or format, as long as you give appropriate credit to the original author(s) and the source, provide a link to the Creative Commons license, and indicate if changes were made.
In cases where reviewers are anonymous, credit should be given to 'Anonymous Referee' and the source.
The images or other third party material in this Peer Review File are included in the article’s Creative Commons license, unless indicated otherwise in a credit line to the material. If material is not included in the article’s Creative Commons license and your intended use is not permitted by statutory regulation or exceeds the permitted use, you will need to obtain permission directly from the copyright holder.
To view a copy of this license, visit https://creativecommons.org/licenses/by/4.0/

Reviewer #1 (Remarks to the Author):

R1: The manuscript reports a free-space mid-infrared silicon membrane metasurface with electrically and optically tunable resonant response. While the device is carefully fabricated and experimentally characterized, the work primarily represents an incremental engineering refinement of previously reported structures and tuning mechanisms. Moreover, the physical description of the tuning processes lacks conceptual precision, further weakening the manuscript's claims of novelty and significance. Overall, the work does not meet the standards required for publication in Nature Communications.

Our response:

We thank the Reviewer for their time to assess our manuscript and appreciate the opportunity to clarify the scope, conceptual framing and technical contributions of our work. We believe that the revisions outlined below will more clearly convey the manuscript's significance for the field of mid-IR photonics.

Below we provide our point by point responses to the Reviewer's comments and indicate how the manuscript has been revised accordingly.

Major Concerns

R1.1

The central device platform—a free-standing (or membrane-type) silicon metasurface operating under free-space mid-infrared excitation—has already been established in prior literature. In particular, free-standing silicon membrane metasurfaces supporting high-Q resonances (including qBIC-type modes) in the mid-infrared have been demonstrated and analyzed in detail in recent high-impact publications (e.g., Nature Communications 15, 1–9 (2024), DOI: 10.1038/s41467-024-54284-0).

In this context, the structural design presented here does not constitute a new class of photonic system, but rather a variation within an already well-explored design space.

Our response:

We thank the Reviewer for raising this point. Indeed, free-standing silicon membrane metasurfaces have been explored for photonic applications. In the mid-IR, however, until very recently, all-dielectric designs were almost exclusively based on Ge or Si resonators on calcium fluoride substrate. This is largely motivated by the main application of mid-IR photonic structures, surface-enhanced infrared absorption spectroscopy (SEIRA) because the associated analyte deposition protocols often rely on spin-coating or biofunctionalization in liquid, which require more robust substrates. The latest works (10.1038/s41467-024-54284-0, which the Reviewer mentions above, as well as a more recent work from this group that we cited in our original manuscript https://doi.org/10.1364/OPTICA.549393), are exclusively focused on well-studied SEIRA application for biochemical sensing and strong light-matter coupling regimes. We note that in these studies, to preserve the membranes, the authors utilized custom analyte deposition protocols that involve evaporation because of the poor compatibility of the fragile free-standing membranes with conventional biofunctionalization steps.

In contrast, our paper focuses on a different application and reports the first use of high-Q silicon membranes for **fast free-space mid-IR light modulation**. The performance metrics and optimization criteria of our application are very different from those reported for SEIRA. Firstly, we demonstrate resonant modes with considerably higher (~4x, 2965 vs 722) measured Q-factors compared to the previous works in mid-IR, which is critical for achieving low-power optical modulation (e.g. 10.1038/s41467-024-54284-0). Secondly, we focus on maintaining **high absolute amplitude contrast** (~50-70%) of the resonances, which is often disregarded in BIC metasurface designs. Thirdly, we demonstrate **dual-mode active tunability** (electro-thermal and ultrafast all-optical). This is again different from the previous work mentioned by the Reviewer in their following comment below, R1.6 (e.g., Russell et al. Laser & Photonics Reviews (2025), DOI: 10.1002/lpor.202400853), which uses only slow thermal tuning with external heaters.

Finally, our work introduces and experimentally demonstrates a novel metasurface design that simultaneously supports ultra–high-Q resonances and remains fully compatible with scalable fabrication techniques, in particular, deep-UV lithography (DUVL). This enables low-cost, wafer-scale manufacturing without compromising optical performance. In contrast, prior studies have predominantly relied on either conventional geometries, such as tilted ellipses, or other designs incorporating smaller features that are challenging to fabricate reliably with DUVL and other wafer-scale approaches and are therefore not suitable for large-scale production (e.g. 10.1364/OPTICA.549393, 10.1038/s41467-024-54284-0). As a result, earlier works used slow and expensive electron-beam lithography, which inherently limits the scalability and practical application potential of such metasurfaces.

ACTIONS TAKEN:

We revised the introduction part of the manuscript to highlight more clearly the unique aspects of our work and the implemented membrane metasurface design on multiple locations as follows:

non-radiative losses[9], limiting the achievable quality factors to $Q \approx 200$. ~~Regarding tunability~~ Very recently, free-standing silicon membrane metasurfaces were explored in the mid-IR as a route to higher Q-factors, as they eliminate lossy substrates and reduce non-radiative losses [37, 58]. These membrane structures have primarily been studied in the context of surface-enhanced infrared absorption (SEIRA) and strong light-matter coupling and involved fully static operation or slow thermal tuning.

By uniting high resonance Q-factors with strong amplitude contrast, efficient dual-mode tunability and wafer-scale fabrication, our silicon membrane metasurfaces address the important bottlenecks in mid-IR photonics technologies. This paves the way for CMOS-compatible, active devices for applications in ~~chemical and biological sensing,~~ high-speed and low-power free-space communications, ~~thermal emission management, and quantum optics~~dynamic control of thermal emission, as well as chemical and biological sensing with enhanced spectral selectivity and sensitivity. In quantum optics, the combination of high-Q resonances with both electrical and optical tunability can enable applications such as cavity-enhanced mid-IR single-photon sources, on-chip quantum spectroscopy of molecular vibrations, and tunable generation of entangled photon pairs. Furthermore, our developed platform opens new avenues for future advances in dynamically reconfigurable photonic systems.

R1.2

The manuscript emphasizes both electrical and optical tuning. However, both tuning routes rely on well-known material responses of silicon:

Electrical tuning proceeds via electro-thermal heating followed by the thermo-optic effect.

Optical tuning is mediated by optically generated charge carriers, which modify the refractive index and absorption through free-carrier dispersion and absorption, often accompanied by secondary heating.

Thermo-optic tuning of semiconductor metasurface resonators, including silicon, has been systematically studied and reviewed, with detailed discussions of resonance shifts, linewidth evolution, and quality-factor dependence on temperature (see Lewi et al., Nanophotonics 8, 331–338 (2019), DOI: 10.1515/nanoph-2018-0178).

Similarly, photoinduced modulation in silicon metasurfaces—where optically generated carriers lead to simultaneous spectral shift and Q variation—has been explicitly reported in recent literature (e.g., Tognazzi et al., Advanced Photonics 5, 066006 (2023), DOI: 10.1117/1.AP.5.6.066006).

Our response:

We thank the reviewer for bringing up these points and allowing us to clarify the contributions of our work. We agree that the explored tuning mechanisms rely on well-established material responses of silicon. However, the novelty of our platform is in the adaptation of these mechanisms to mid-IR photonics, where devices for active light control and routing are very scarce. In fact, the first work that the reviewer mentions (Tognazzi et al., DOI: 10.1117/1.AP.5.6.066006) studies photoinduced reflectivity modulation in silicon metasurfaces within the near-infrared telecom wavelength range (1300-1400 nm), not in the Mid-IR spectrum as we focus in our paper. The other work that the reviewer mentions (Lewi et al., DOI: 10.1515/nanoph-2018-0178) studies only the thermal tuning process with silicon metasurfaces at NIR (telecom) range and with silicon and germanium single particle resonators at Mid-IR. This study does not incorporate an on-chip electro-thermal tuning as we utilize in our work. In addition, it does not exploit the all-optical modulation process in Mid-IR with metasurfaces, hence its modulation is not even comparable to the ultra-fast modulation speed that we report in our study.

To position the prospect of our study in the broader context, it is important to highlight that the Mid-infrared photonics field has been historically driven by absorption spectroscopy applications and relied on sophisticated bulk-optic instrumentation that compensated for the lack of efficient sources and detectors. Currently, the landscape in the field is rapidly changing due to maturing of quantum cascade laser technology and significant progress in mid-IR emission and detection concepts, that are expanding possible applications beyond static sensing and toward dynamic imaging, adaptive spectroscopy, active thermal emission control, etc. These emerging applications require the same level of optical control that is routine in the visible and near-IR (telecom) ranges such as fast intensity and phase modulation, spatial light modulation, beam steering and dynamically tunable, CMOS-compatible active components with high spectral selectivity. Yet, the equivalent mid-IR optical components are still immature, which provides a strong motivation for our work.

ACTION TAKEN:

We cited the relevant papers suggested by the reviewer and made a revision of the introduction briefly mentioning the important gaps and shortcomings that our results address:

Mid-infrared (mid-IR) photonics have the potential to greatly benefit from both the high Q-factor modes and active tuning, which can be utilized for enhanced detection of chemical and biological compounds[11], spectroscopy, and efficient high harmonic generation[35]. However, The field has been historically driven by absorption spectroscopy applications and relied on sophisticated bulk-optic instrumentation that

compensated for the lack of efficient sources and detectors. Currently, the landscape is rapidly changing due to maturing of quantum cascade laser technology[36] and significant progress in mid-IR emission and detection concepts that are expanding possible applications beyond static sensing and toward dynamic imaging[37], adaptive spectroscopy, active thermal emission control[38], and more. These emerging applications require the same level of optical control that is routine in the visible and near-IR (telecom) ranges such as fast intensity and phase modulation, spatial light modulation, beam steering and dynamically tunable, CMOS-compatible active components with high spectral selectivity. Notably, despite the significantly relaxed fabrication requirements for mid-IR metasurfaces as compared to their visible/near-

…

strate an on-chip device that features strong electro-thermal modulation induced by a CMOS-compatible voltage of 5 V sustaining > 50% absolute signal variation with frequencies up to 14.5 kHz. This platform with integrated, actively modulated heaters enables significantly faster and more precise control compared to the external thermal heating methods[48]. In the second, we achieve high-speed (sub-GHz) all-optical modulation using the ultrafast optically induced charge carrier generation in silicon.

…

(sub-GHz) all-optical modulation using the ultrafast optically induced charge carrier generation in silicon. Sharp resonances combined with strong sensitivity of the optical response of silicon to the generated charge density in the mid-IR range allow for efficient modulation at pump fluences almost 2 orders of magnitude lower than for similar solutions in the near-infrared and visible spectral ranges[63]. Importantly,

R1.3

The manuscript repeatedly employs the term electro-optic to describe the electrically driven tuning process. Conventionally, electro-optic effects refer to field-driven refractive-index modulation (e.g., Pockels or Kerr effects).

In the present work, the electrical control modifies the optical response indirectly through heating and carrier-related processes, rather than through a direct electric-field–induced refractive-index change. Referring to this mechanism as “electro-optic” therefore conflates actuation pathways with optical modulation mechanisms and introduces conceptual ambiguity.

Our response:

We thank the reviewer for bringing up this point regarding terminology. We agree that, in a strict physical sense, *electro-optic effects* refer to field-driven refractive-index modulation (e.g., Pockels or Kerr effects), whereas in our devices the electrical bias modifies the optical response indirectly via electro-thermal processes.

At the same time we would like to highlight that the use of the term *electro-optic* for devices relying on Joule heating effects is also common. Examples can be found in the literature, for instance, in this review article: Junge et al. "The rise of electrically tunable metasurfaces" Science Advances 10, 34 (2024) 10.1126/sciadv.ado8964.

Based on the reviewer's suggestion, to avoid ambiguity, we revised the manuscript to replace "electro-optic" with a more precise term (electro-thermal) where appropriate.

ACTIONS TAKEN:

Manuscript text and Figure content and captions were revised to replace "electro-optical" with the more appropriate term "electro-thermal"

R1.4

Given that both the device architecture and tuning mechanisms have clear precedents, the primary contribution of the manuscript lies in engineering refinement, such as geometry optimization or resonance shaping.

While such refinements are valuable from an implementation standpoint, they do not establish a new physical regime, scaling law, or modulation paradigm.

Our response:

We would like to emphasize that translating the established concepts for light control in the visible/near-IR spectrum into the mid-IR is not a straightforward wavelength scaling task. On one hand, the mid-IR regime imposes severe constraints on material platforms due to the onset of absorption. On the other hand, relevant interaction mechanisms are stronger (e.g., free-carrier and thermo-optic responses, access to phonon/polariton resonances, etc) and critical dimension requirements for lithography are more relaxed. In combination, these aspects create an appealing research opportunity for new Mid-IR device architectures with larger modulation depth and lower drive power. Therefore, we consider our results not merely an engineering refinement, but an important advancement that establishes membrane metasurfaces as a platform for mid-IR devices for active light control and routing.

ACTIONS TAKEN:

We revised the introduction part of the manuscript to include our arguments on the novelty of our results, in addition to what is discussed above in our response to R1.1-R1.2:

From the tunability perspective, reconfigurable metasurfaces based on chalcogenide phase-change materials enable strong modulation depth but are limited to low switching speeds, as their tuning mechanism relies on heating and cooling sequences [42–45]. Similar restrictions arise in devices relying on gas absorption [46], intercalation of ions [47] or thermal tuning [48]. On the other hand, electrostatic tuning [48, 49]. Electrostatic tuning of free-carrier–based platforms including graphene[50, 51] , ITO [52], and carbon nanotube films and metamaterials [53–55] can potentially provide high (sub-GHz) modulation frequenciesin metasurfaces based on graphene[50, 51] and ITO [52]. However, the plasmonic nature of these structures results in even lower Q-factors, requiring high operating voltages to achieve reasonable modulation depth[56] and rendering them poorly compatible with CMOS standardsthese approaches rely on plasmonic or intraband responses associated with increased optical losses and typically require large carrier densities to achieve substantial modulation depth, which often translates into elevated operating voltages and reduced spectral selectivity[56], complicating CMOS-level integration. This highlights the need for that translating the well-developed tuning mechanisms for light control into the mid-IR is not a straightforward wavelength scaling task. Therefore, the development of alternative material platforms and design strategies for metasurfaces that can simultaneously support high-Q resonances and efficient, high-speed,

low-power tunability in the mid-IR while maintaining compatibility with wafer-scale manufacturingfor real-world , is critically important for practical applications.

R1.5

Although relatively high quality factors are reported, the tuning process—whether electrical or optical—is accompanied by continuous spectral shifting of the resonance, during which both the resonance wavelength and the Q factor evolve.
As a result, the high-Q resonance does not represent a stable operating point during modulation. The observed intensity variation largely arises from the resonance moving across a fixed probe wavelength, limiting the functional benefit of achieving a high Q factor in this configuration.

Our response:

We thank the opportunity to clarify the operational implementation of our device. We agree that the modulation mechanism that we present involves the evolution of both resonance wavelength and the Q factor. However, this does not preclude the existence of a well-defined and practically useful operation point.

The modulation in our device arises from controlled tuning of the resonance relative to a fixed probe wavelength. In this context, the role of a high-Q resonance is not to serve as a static operating point, but rather to provide a steep spectral response. This steepness directly enhances modulation efficiency because without a sufficiently high Q factor, a larger driving power would be required to induce a spectral shift comparable to the resonance linewidth and achieve meaningful intensity modulation. We illustrated this aspect experimentally by demonstrating modulation at pump powers 2 orders of magnitude lower than what is typically needed for analogous visible/near-IR structures, and achieving a large electro-thermal signal amplitude modulation for small changes of

the membrane temperature. This feature can be used, for example, for efficient low-power modulation of narrowband mid-IR quantum cascade lasers. We also want to add that our platform allows flexibility for modulation at lower Q-factor values (thus larger bandwidths), if needed for a particular use case. In summary, the high-Q resonance plays a central functional role by reducing required tuning amplitude (and thus driving power) and enhancing modulation contrast, rather than serving as a static spectral feature.

ACTION TAKEN:
We highlighted the specific use cases enabled by efficient low-power tuning of high Q-factor resonances in the outlook of the paper:

address the important bottlenecks in mid-IR photonics technologies. This paves the way for CMOS-compatible, active devices for applications in ~~chemical and biological sensing,~~ high-speed and low-power free-space communications, ~~thermal emission management, and quantum optics~~dynamic control of thermal emission, as well as chemical and biological sensing with enhanced spectral selectivity and sensitivity. In quantum optics, the combination of high-Q resonances with both electrical and optical tunability can enable applications such as cavity-enhanced mid-IR single-photon sources, on-chip quantum spectroscopy of molecular vibrations, and tunable generation of entangled photon pairs. Furthermore, our developed platform opens new avenues for future advances in dynamically reconfigurable photonic systems.

R1.6
Even when evaluated as an engineering implementation, the reported performance does not clearly surpass existing approaches. For example, thermo-optically tunable mid-infrared silicon metasurface filters have already demonstrated controlled, reversible spectral tuning with well-defined application contexts (Russell et al., Laser & Photonics Reviews (2025), DOI: 10.1002/lpor.202400853).

By comparison, the present manuscript does not demonstrate superior modulation depth, speed, energy efficiency, or stability. Intrinsic limitations associated with thermal diffusion and carrier dynamics further constrain achievable modulation speed and energy consumption, and no evidence is provided that these limits are meaningfully improved.

Our response:
We cannot agree with the Reviewer on this comment. In fact, in our paper, we demonstrate following features:
1. Showed far superior modulation speed, especially compared to the solutions based on external heaters, such as Russel et al. (10.1002/lpor.202400853) mentioned by the Reviewer or a more recent paper by Kuruoglu et al. (https://doi.org/10.48550/arXiv.2506.10115). In fact, neither of these papers report the actual modulation speed, which is a severe bottleneck for external heater solutions with poor thermal diffusion. In our work, we explicitly demonstrate the electro-thermal modulation speed and its improvement for direct and local membrane heating designs, as well as estimate the recovery rate for all-optical modulation, which both represent a meaningful improvement over the reported active mid-IR metasurfaces.

2. Achieved the highest quality factors for the free-space mid-IR optics thanks to our metasurface design and the fabrication quality, as well as by our optimized experiment configuration, which can be adopted by others in the field in further research.
3. Showed experimentally an important advantage of the all-optical modulation approach based on charge carrier generation for mid-IR photonics through demonstration of a large modulation contrast with record-low pump fluences (~4 uJ/cm^2), which is two orders of magnitude smaller than what was so far reported for visible and near-infrared metasurfaces, including the work by Tognazzi et al. 10.1117/1.AP.5.6.066006 (250 uJ/cm^2) mentioned by the Reviewer in their previous comment above, R1.2.

ACTIONS TAKEN:
We revised the manuscript to highlight the unique advantages of our platform and the significant advancements over previous work as follows.

Alternative mid-IR tuning platforms:

quences [42–45]. Similar restrictions arise in devices relying on gas absorption [46], intercalation of ions [47] or thermal tuning [48]. On the other hand, electrostatic tuning [48, 49]. Electrostatic tuning of free-carrier–based platforms including graphene[50, 51] , ITO [52], and carbon nanotube films and metamaterials [53–55] can potentially provide high (sub-GHz) modulation frequenciesin metasurfaces based on graphene[50, 51] and ITO [52]. However, the plasmonic nature of these structures results in even lower Q-factors, requiring high operating voltages to achieve reasonable modulation depth[56] and rendering them poorly compatible with CMOS standardsthese approaches rely on plasmonic or intraband responses associated with increased optical losses and typically require large carrier densities to achieve substantial modulation depth, which often translates into elevated operating voltages and reduced spectral selectivity[56], complicating CMOS-level integration. This highlights the need for that translating the well-developed tuning mechanisms for light control into the mid-IR is not a straightforward wavelength scaling task. Therefore, the development of alternative material platforms and design strategies for metasurfaces that can simultaneously support high-Q resonances and efficient, high-speed, low-power tunability in the mid-IR while maintaining compatibility with wafer-scale manufacturingfor real-world , is critically important for practical applications.

Advantages over external heater solutions:

strate an on-chip device that features strong electro-thermal modulation induced by a CMOS-compatible voltage of 5 V sustaining > 50% absolute signal variation with frequencies up to 14.5 kHz. This platform with integrated, actively modulated heaters enables significantly faster and more precise control compared to the external thermal heating methods[48]. In the second, we achieve high-speed (sub-GHz) all-optical modulation using the ultrafast optically induced charge carrier generation in silicon.

Superior optical tuning efficiency:

ulation using the ultrafast optically induced charge carrier generation in silicon. Sharp resonances combined with strong sensitivity of the optical response of silicon to the generated charge density in the mid-IR range allow for efficient modulation at pump fluences almost 2 orders of magnitude lower than for similar solutions in the near-infrared and visible spectral ranges[57]. Importantly, we demonstrate full

Wafer-scale fabrication:

the near-infrared and visible spectral ranges[57]. Importantly, we demonstrate full compatibility of the design with wafer-scale manufacturing using deep-UV lithography (DUVL) without compromising optical performance compared to chip-scale electron beam lithography. Our results establish membrane metasurfaces as a versatile platform for active mid-IR light routing that we envision for applications in chemical and biological sensingscalable platform for high-speed, high-contrast, low-power active light routing in the mid-IR, where efficient and actively tunable free-space photonic devices remain very scarce. We envision applications of our platform in free space communications, thermal emission management, chemical and biological sensing, as well as in the emerging fields of mid-IR quantum opticsand quantum communications.

Aspects of the measurement setup

change rapidly with the incidence angle. As a result, standard FTIR microscopes with reflective Cassegrain objectives or high NA refractive objectives are not well suited for characterization of high-Q modes. For accurate measurements of our membrane, we developed a custom FTIR microscope accessory with spatial filtering of optical signal in the back focal plane, as illustrated in Figure 2f. It allowed us to limit the angular spread of the incident mid-IR light down to several degrees, corresponding to an By closing the size of the filter aperture down to 1 mm we reduced the effective NA of the optical system down to 0.02, which allowed us to isolate the response from near-normal angles of incidence that correspond to the highest mode Q-factors. Figure 2d shows the transmission spectrum of the metasurface with a hole ellipticity of $\varepsilon = 1.08$ fab-

Outlook and applications

address the important bottlenecks in mid-IR photonics technologies. This paves the way for CMOS-compatible, active devices for applications in chemical and biological sensing, high-speed and low-power free-space communications, thermal emission management, and quantum opticsdynamic control of thermal emission, as well as chemical and biological sensing with enhanced spectral selectivity and sensitivity. In quantum optics, the combination of high-Q resonances with both electrical and optical tunability can enable applications such as cavity-enhanced mid-IR single-photon sources, on-chip quantum spectroscopy of molecular vibrations, and tunable generation of entangled photon pairs. Furthermore, our developed platform opens new avenues for future advances in dynamically reconfigurable photonic systems.

Reviewer #2 (Remarks to the Author):

R2: This manuscript presents a suspended silicon membrane platform for the mid-infrared (5–6 μm) that utilizes qBIC resonances to achieve high Q-factors (~2500). The authors demonstrate two modulation schemes: electro-thermal tuning and ultrafast all-optical modulation. This work aims to overcome substrate loss limitations to create a scalable, CMOS-compatible modulator. The suspended silicon platform is technically sound, the reported Q-factors are impressive, and the dynamic modulation is considered effective in this field. The manuscript is logically organized, and the conclusions are supported by the presented results. The manuscript can be further strengthened by addressing the following comments.

Our response:
We thank Reviewer #2 for the positive assessment and constructive suggestions. We address each point below.

R2.1
The manuscript claims "record-high measured Q-factors up to 3000" in the abstract. However, it is just one data point in Figure 2e. The transmission spectrum corresponding to the highest reported Q-factor should be provided. The claim is also inconsistent in the manuscript. In Table 2 in the Supporting Information, the authors use 2500 for this work to be compared with others.
Our response:
We thank the reviewer for noting this point. In the fabricated devices from our later batch, we achieve the measured Q-factors approaching 3000 for devices with a low asymmetry parameter. The initial version of Figure 2 contained a representative transmission spectrum of a device from our initial batch that had a slightly lower Q-factor. During the course of the originally submitted work, we refined our fabrication procedure, in particular, using elliptical curve fracturing of the e-beam exposure step, and from the devices of the later batch we were able to obtain measured Q-factors as high as 2965 with modulation of up to 55%, which represents a >4x (2965 vs ~720-730) improvement over the recent works on mid-IR membrane metasurfaces [10.1038/s41467-024-54284-0, https://doi.org/10.1364/OPTICA.549393].

To avoid the confusion with different numbers, we updated Fig. 2 with the spectrum of the device from the later batch reaching a Q-factor of 2965, which was used in thermo-optical tuning experiments (Fig. 3). We also updated Table 2 of the Supporting Information accordingly.

<u>ACTIONS TAKEN:</u>
We updated Fig.2d transmission spectra for the device with the highest measured Q-factor.

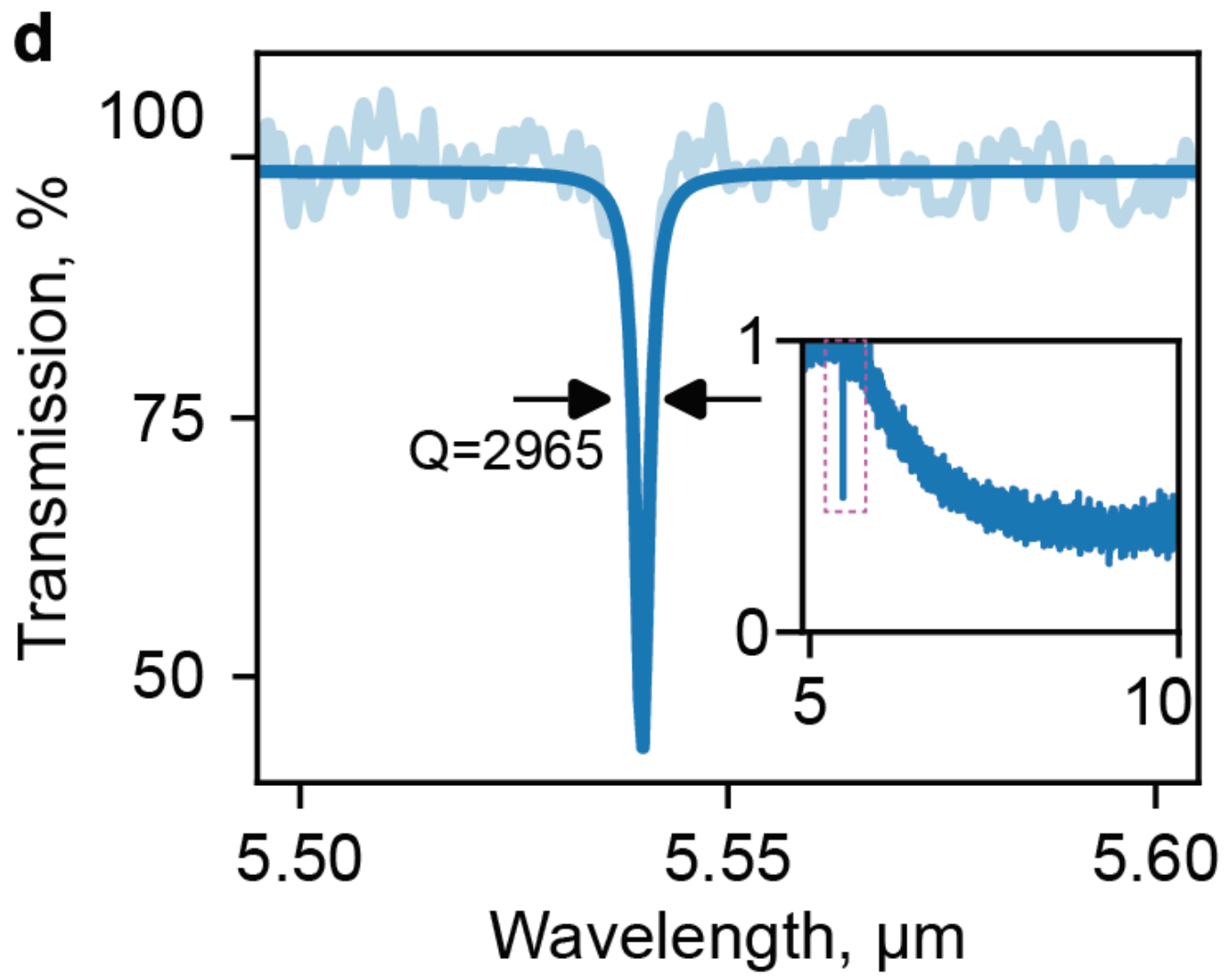

Revised Fig. 2d of the main manuscript.

R2.2
The authors use phosphorous doping to improve heating efficiency. However, there is no comparison data demonstrating how the doping affects q-factors, i.e., the same device before and after doping.

Our response:
We thank the Reviewer for bringing up this important discussion. Indeed, high doping levels contribute to the increase of the optical loss (as well as to a change of the real part of the refractive index) and therefore can lead to modification of the Q-factor, spectral position, and modulation amplitude of the resonance. In reality, there is a trade-off between the conductivity and, consequently, heating efficiency of the membrane, and the quality of the resonance.
In our experiments, we observed no difference between the resonances of the membrane without doping and the one that was exposed for 1 minute. Starting from 2 min exposure time, both Q-factor and the amplitude of resonance begin to drop (see Figure R1). As 1 min doping time is enough to improve the conductivity of the membrane without affecting the resonance, this exposure time was used for demonstration of the electro-thermal modulation in the main manuscript.

<u>ACTIONS TAKEN:</u>
To illustrate the influence of doping level on the optical properties of the membrane, we added the result in Figure R1 in the Supplementary information (Section 3) that shows the dependence of Q-factor and modulation amplitude of the main resonance on the exposure time during the doping process.

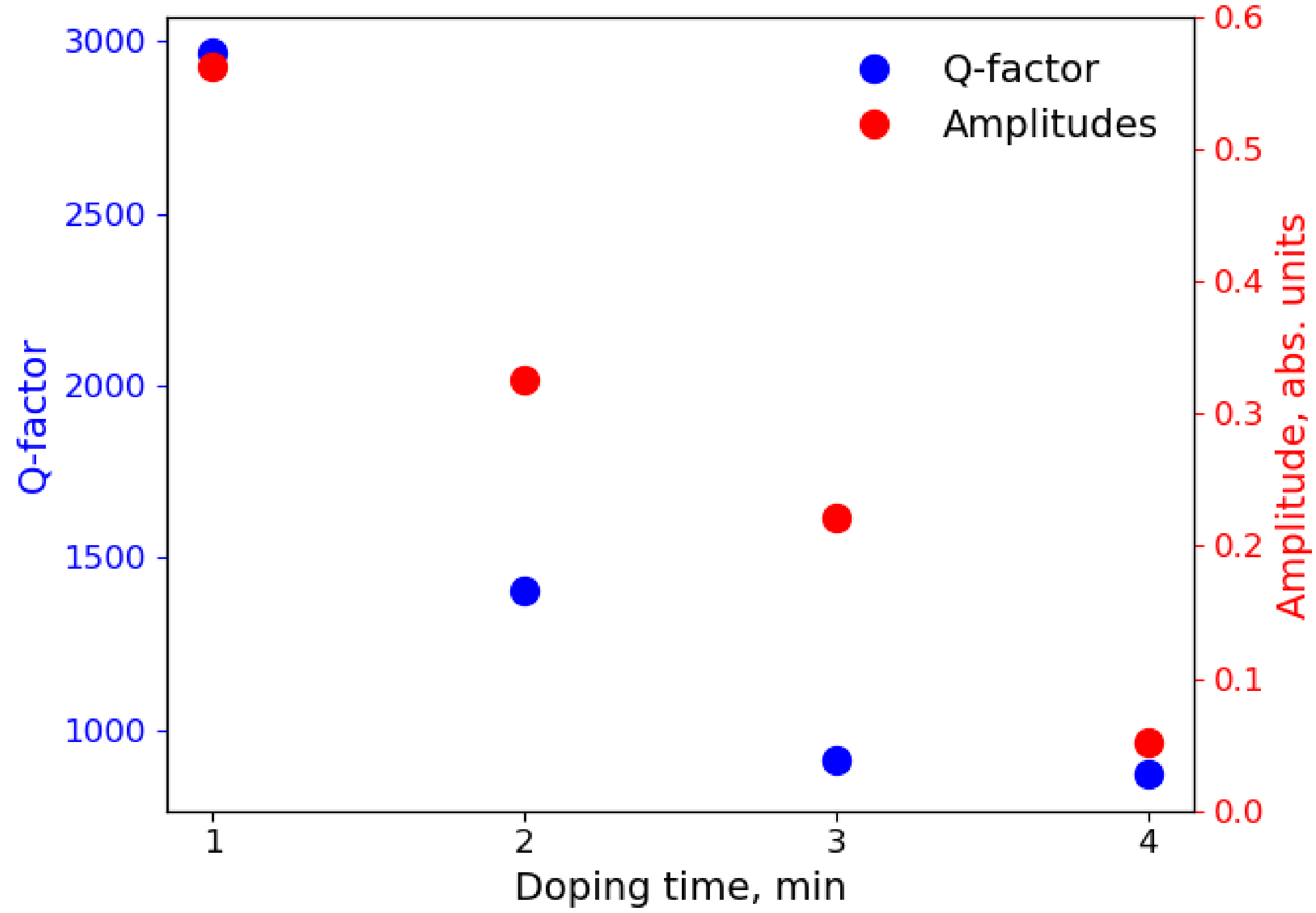

Figure R1. Dependence of the measured Q-factor and amplitude membrane metasurfaces with varying doping time.

R2.3

The manuscript claims wafer-scale fabrication compatible with the demonstration of direct laser writing. However, there is only one datapoint for the DLW sample. It is not clear if the SEM image of Figure 2c is from DLW or EBL. Also, no consistency analysis, design/device mismatch, or imperfection analysis is performed for DLW samples. This is critical for feature sizes close to equipment limitations.

Our response:

We thank the reviewer for raising this relevant point. Following this suggestion, during the revision process, we have established a wafer-scale fabrication protocol using deep-UV (DUV) lithography that offers superior exposure uniformity and shape fidelity compared to direct laser writing (DLW). By adjusting the exposure dose of DUV, we achieved exact matching of the parameters between the design and the fabricated structures. Consequently, our optimized DUVL protocol provided excellent matching with EBL structures in terms of the range of achievable Q-factors and the modulation amplitude. We also performed new experiments for consistency analysis, which is included in the updated Figure 2e by showing data measured from membranes fabricated by both DUV (triangles) and EBL (hollow circles) methods, see below. Furthermore, we did not observe any detectable difference in the SEM images of DLW and DUV structures (lower Q-factor and amplitudes of resonances for DLW structures

were mainly related to the small-scale exposure dose variations). Therefore, we kept the original images of a DLW wafer in Fig. 2a,b for the purposes of illustration and replaced Fig. 2c with the SEM image of a DUV membrane. Figure caption is revised accordingly.

ACTIONS TAKEN:

We have updated Figure 2c,e with the results from structures fabricated with deep-UV lithography. We also specified the source of the SEM data in the caption of Figure 2.

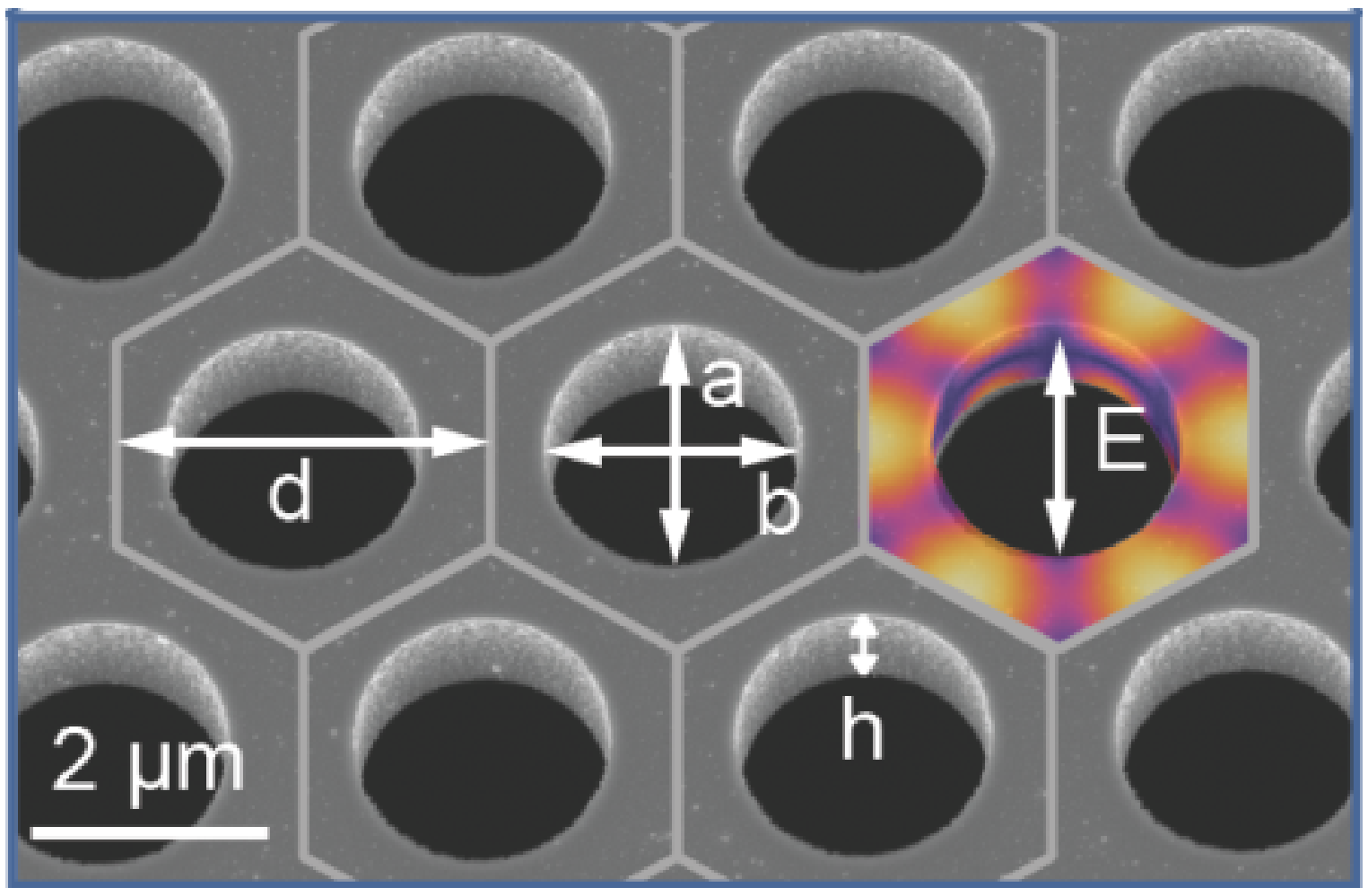

Revised Figure 2c caption: “SEM image of a DUV-fabricated Si membrane metasurface. Colorful overlay on one of the unit cells shows the calculated distribution of the electric field at the qBIC resonance excited with linearly polarized plane wave E at λ_{res} = 5.54 μm.”

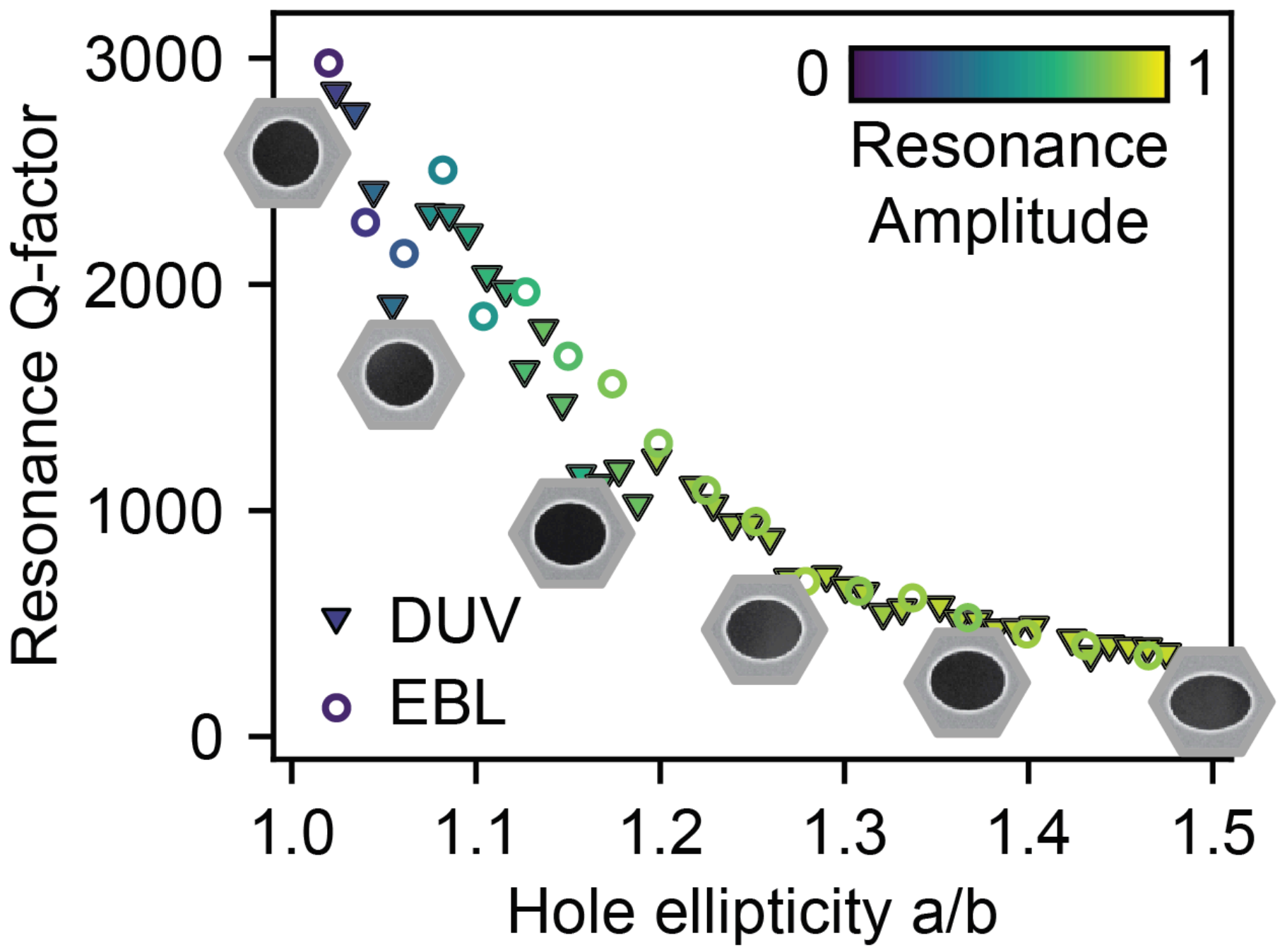

Revised Figure 2e. “Dependence of the measured Q-factor for EBL (hollow circles) and DUV (solid triangles) written membrane metasurfaces with varying hole ellipticity.

Color of the symbols encodes the measured resonance amplitude. The insets show SEM images of the corresponding unit cell."

R2.4

Line 119, "we keep the hole area constant at A=1.1um2". However, Figure 2c indicates that the hole radius is larger than 1 μm. Wrong scale bar?

Our response:

We thank the Reviewer for careful reading and highlighting this discrepancy. Indeed, we simply forgot to include the factor of pi in our calculation. The correct area is: A=3.5μm².

ACTIONS TAKEN:

We corrected the number in the revised manuscript.

R2.5

It is not clear how the infrared transmission spectra in Figures 5d and 5h are obtained. As far as the reviewer knows, the KYMERA-193i-B2 spectrograph is for Vis-NIR. A detailed description of the MIR measurement is helpful for the reader.

Our response:

We thank the reviewer for this comment and allowing us to describe our experimental set-up better. The transmission spectra are measured on the commercial transient absorption spectroscopy system (HARPIA-TA) in MIR configuration from Light Conversion. For spectroscopic detection, the system uses 150 lines/mm MIR diffraction grating that projects the light onto a LN-cooled point MCT detector (Infrared Associates MCT-13-1.0). Spectrally resolved measurements are performed by scanning the grating angle.

ACTIONS TAKEN:

We revised the Methods section to include more detailed information on the MIR time-resolved measurements as follows:

were split for the pump and probe beams. Each beam was individually tuned using an optical parametric amplifier (Orpheus-HP for the pump and Orpheus-One-HP for the probe). Both beams were focused on the sample, which was positioned orthogonally to the probe beam. ~~The infrared~~ Infrared transmission spectra were collected using an Andor KYMERA-193i-B2 spectrograph, equipped with a 150 mm^{-1} mid-IR diffraction grating and a point MCT detector (Infrared Associates MCT-13-1.0). For the average (chopped) measured pump power of 1.2 mW and an approximate beam area of 1 mm^2, we estimate a fluence of 4.8 $\mu J\,cm^{-2}$.

Reviewer #3 (Remarks to the Author):

R3: This paper reports a mid-infrared optical light modulator based on high-Q silicon metasurfaces. The authors demonstrate thermally and optically tunable transmission

resonances around a wavelength of 5 µm. Very high quality factors are achieved using bound-state-in-the-continuum (BIC)–type resonances.

The paper is well written, and the results are sound and interesting. I recommend this paper for publication in Nature Communications, subject to minor revisions.

I have a few minor comments that may help to improve the manuscript:

Our response:
We thank Reviewer #3 for the positive evaluation and helpful suggestions.

R3.1
The physical origin of the BIC resonance is not discussed in sufficient detail. A clearer explanation of the nature of this resonance and the mechanism leading to the high-Q response would strengthen the paper.

Our response:
We thank the reviewer for this valuable suggestion. In the revised manuscript, we provided an extended discussion on the origin of the observed resonance.

ACTIONS TAKEN:
The revised section describing the design choices now reads as follows:

Our membrane metasurfaces are based on CMOS-grade silicon-on-insulator substrates ~~using~~ that are compatible with high-throughput wafer-scale processes. ~~A metasurface consists of a $h = 1\,\mu m$ thick single-crystalline silicon membrane, perforated by holes arranged in a hexagonal lattice with a period of $d = 3.3\,\mu m$ (for the fabrication details, see Methods). We numerically optimize the structure parameters so that for circular shaped holes, the metasurface supports a~~ To incorporate a high-Q mode with a linewidth that can be easily controlled with design parameters, we utilize the well-established concept of symmetry-protected bound ~~state~~ states in the continuum~~in the 5-6 µm spectral range[59, 60]. This~~ [58]. As a base design, we choose the highly symmetric hexagonal (C_{6v}) lattice of circular holes in a silicon slab to ensure robustness of the resulting suspended membrane. The non-radiative ~~state can then~~ BIC states supported in such a lattice can be transformed into ~~a mode with a~~ modes with finite Q~~-factor~~ -factors by breaking the symmetry using the quasi-BIC principle[16]

according to the established selection rules[59]. In our case, we ~~control the radiative losses of the qBIC mode~~ reduce the symmetry of the structure to C_{2v} by introducing ellipticity to the holes: $\varepsilon = \frac{a}{b}$ (Figure 2c).

Using this design principle, we numerically optimize the structure parameters to rescale the BIC condition to the 5-6 µm spectral range using the recent paper[60] as a reference. Fixing the design to a commercially available base silicon layer thickness of $h = 1\,\mu m$, we end up with a structure with a lattice period of $d = 3.3\,\mu m$. To tune the Q-factor without a considerable spectral shift of the resonance, when changing the ellipticity we keep the hole area constant at ~~$A = 1.1\,\mu m^2$~~ $A = 3.5\,\mu m^2$.

R3.2
The experimental method used to measure the BIC resonance at normal incidence is not sufficiently clear. In particular, the authors should explain why a spatial filter is required and discuss whether the filter could introduce any artificial resonance effects.

Our response:
We thank the reviewer for this comment and allowing us to provide more details for our experimental method. In the literature, it is well established that the radiative losses of symmetry-protected BIC and q-BIC modes rapidly increase for off-Γ excitation due to additional symmetry breaking introduced by oblique incidence geometry, leading to the observation of broader resonances. Focussed light beams utilized in microscopy measurements contain angles of incidence up to the numerical aperture of the optics involved, which leads to simultaneous detection of high-Q near-normal components as well as lower-Q oblique components. Another effect contributing to the broadening of the measured resonances is the angular dispersion of the optical modes of the membrane, i.e. the dependence of their resonance frequency on the angle of incidence. We demonstrate it explicitly with our mid-IR back focal plane measurements in Fig. 3.

By utilizing a spatial filter in our setup, schematically shown in Figure 2f, we limit the effective numerical aperture of our system down to ~0.02, which allows us to accurately quantify the Q-factor of the resonance at normal incidence. For the same reason, we use a spatial filter in the back focal plane of the setup for electro-thermal modulation experiments, as it allows us to characterize the performance of the membrane for normally incident light. At the same time, the physical size of the opening of the spatial filter is ~1 mm, which prevents any possible artifacts from e.g. scattering or diffraction on its edges.

<u>ACTIONS TAKEN:</u>
We expanded the discussion on page 4 of the revised manuscript, describing the purpose of using the spatial filter in the back focal plane of the setup:

The crystallinity of the metasurface material and the absence of a substrate that can induce additional optical losses make the silicon membrane-based metasurfaces ideal for achieving ultra-high Q-factors in the mid-infrared range. However, direct observation of these high Q-factors is also largely dependent on the measurement procedure, as the linewidth and spectral position of the qBIC modes tend to change rapidly with the incidence angle. As a result, standard FTIR microscopes with reflective Cassegrain objectives or high NA refractive objectives are not well suited for characterization of high-Q modes. For accurate measurements of our membrane, we developed a custom FTIR microscope accessory with spatial filtering of optical signal in the back focal plane, as illustrated in Figure 2f. ~~It allowed us to limit the angular spread of the incident mid-IR light down to several degrees, corresponding to an~~ By closing the size of the filter aperture down to 1 mm we reduced the effective NA of the optical system down to 0.02, which allowed us to isolate the response from near-normal angles of incidence that correspond to the highest mode Q-factors. Figure 2d shows the transmission spectrum of the metasurface with a hole ellipticity of $\varepsilon = 1.08$ fab-

R3.3

It would be beneficial to compare this approach with other infrared-active materials and modulation platforms, such as carbon nanotubes and graphene, to better contextualize the advantages and limitations of the proposed system.

Our response:

We thank the Reviewer for this valuable suggestion. We agree that in the original manuscript, the comparison of our approach was limited to the established infrared-active tuning platforms, including graphene- and ITO-based electrostatically controlled metasurfaces as well as thermally driven phase-change materials. Following reviewer's suggestion, in the revised manuscript, we have expanded the discussion to more clearly position our approach relative to other infrared-active material platforms, particularly graphene and carbon nanotubes.

ACTIONS TAKEN:

The revised section comparing our approach with other infrared-active material and modulation platforms reads as follows:

From the tunability perspective, reconfigurable metasurfaces based on chalcogenide phase-change materials enable strong modulation depth but are limited to low switching speeds, as their tuning mechanism relies on heating and cooling sequences [42–45]. Similar restrictions arise in devices relying on gas absorption [46], intercalation of ions [47] or thermal tuning ~~[48]. On the other hand, electrostatic tuning~~ [48, 49]. Electrostatic tuning of free-carrier–based platforms including graphene[50, 51] , ITO [52], and carbon nanotube films and metamaterials [53–55] can potentially provide high (sub-GHz) modulation frequencies~~in metasurfaces based on graphene[50, 51] and ITO [52]~~. However, ~~the plasmonic nature of these structures results in even lower Q-factors, requiring high operating voltages to achieve reasonable modulation depth[56] and rendering them poorly compatible with CMOS standards~~these approaches rely on plasmonic or intraband responses associated with increased optical losses and typically require large carrier densities to achieve substantial modulation depth, which often translates into elevated operating voltages and reduced spectral selectivity[56], complicating CMOS-level integration. This high-

R3.4

The potential applications of this method should be discussed more concretely. The authors mention possible use in quantum photonics, but this claim is not sufficiently supported and would benefit from clarification or justification.

Our response:

We thank the reviewer for raising this point. By combining high-Q resonances with strong amplitude contrast and dual electrical and optical tunability in a CMOS-compatible wafer-scale platform, our silicon membrane metasurfaces enable low-power modulation and precise spectral control in the mid-IR spectrum. These capabilities support classical applications such as dynamic control of thermal emission, high-speed and low-power free-space optical communication, as well as chemical and biological sensing with enhanced spectral selectivity and sensitivity. In the context of quantum photonics, high-Q

resonators can increase the emission rates of mid-IR quantum emitters, enabling on-chip single-photon generation. Nonlinear processes can be enhanced in high-Q cavities, while tunable metasurfaces allow spectral and temporal shaping of photon pairs. Moreover, fast dual-mode modulation enables dynamic tuning of the emitter–cavity detuning, which is critical for deterministic control of quantum states in on-chip photonic circuits. These points have been incorporated into the revised manuscript.

ACTIONS TAKEN:

The revised paragraph highlighting potential applications more concretely reads as follows:

By uniting high resonance Q-factors with strong amplitude contrast, efficient dual-mode tunability and wafer-scale fabrication, our silicon membrane metasurfaces address the important bottlenecks in mid-IR photonics technologies. This paves the way for CMOS-compatible, active devices for applications in ~~chemical and biological sensing,~~ high-speed and low-power free-space communications, ~~thermal emission management, and quantum optics~~dynamic control of thermal emission, as well as chemical and biological sensing with enhanced spectral selectivity and sensitivity. In quantum optics, the combination of high-Q resonances with both electrical and optical tunability can enable applications such as cavity-enhanced mid-IR single-photon sources, on-chip quantum spectroscopy of molecular vibrations, and tunable generation of entangled photon pairs. Furthermore, our developed platform opens new avenues for future advances in dynamically reconfigurable photonic systems.

Reviewer #4 (Remarks to the Author):

R4: I co-reviewed this manuscript with one of the reviewers who provided the listed reports. This is part of the Nature Communications initiative to facilitate training in peer review and to provide appropriate recognition for Early Career Researchers who co-review manuscripts.

Our response:

We thank Reviewer #4 for their contribution to the review process.

Reviewer #1 (Remarks to the Author):

I thank the authors for their detailed rebuttal and the substantial experimental efforts made during the revision. The implementation of the wafer-scale DUV lithography protocol and the supplementary doping-time optimization data are well received. The technical execution of achieving a measured Q-factor approaching 3000 in a free-standing silicon membrane is highly acknowledged.

However, while the fabrication quality is solid, the manuscript fundamentally remains an incremental technical refinement. Moving an established device architecture to the mid-infrared (mid-IR) spectrum is a valuable engineering application, but it cannot serve as a justification for limited device-level performance and a lack of fundamental physical novelty. Several critical bottlenecks regarding performance and practical viability remain unaddressed:

Our response:

We thank the Reviewer for acknowledging both the extensive additional experimental efforts and the achieved device quality, particularly the reproducibly high Q-factor obtained in a large-scale realization.

We respectfully disagree, however, with the characterization of the work as merely an incremental refinement. While the individual building blocks and underlying principles may be known separately, the novelty of the present work lies in their first-time integration into a single mid-IR modulation platform that is compatible with large-scale fabrication and therefore positioned for technology translation.

Importantly, our work directly demonstrates several unique features of the silicon membrane platform that can enable further advances in mid-IR photonics. Firstly, we demonstrate free-carrier effects that are substantially stronger than in the well-studied visible and near-IR spectral ranges, making them particularly promising for efficient modulation. We believe this capability opens the way toward novel mid-IR modulation schemes that will exploit strong spatial refractive-index gradients induced by carrier injection.

Secondly, we identify the important role of surface recombination dynamics of charge carriers in membrane metasurface architectures. This mechanism enables fast all-optical modulation despite comparatively slow (µs-scale) volumetric recombination, thereby revealing an important and previously underexplored aspect of carrier dynamics in suspended mid-IR silicon structures.

Lastly, we highlight the tradeoff between material absorption induced by doping and resonance Q-factor in the context of efficient electro-thermal modulation. Understanding and balancing these competing effects is essential for achieving practical device operation in the mid-IR regime.

These aspects are particularly important because the vast majority of materials exhibit strong absorption losses in the mid-IR spectral range, severely constraining the design space for low-loss active photonic devices. In this context, the demonstrated silicon membrane platform provides a viable route toward high-amplitude, low-loss modulation, especially in transmission-mode configurations.

For these reasons, we believe the present work represents more than a straightforward spectral extension of existing concepts and approaches. Rather, it establishes a scalable and experimentally validated platform that addresses several key challenges specific to mid-IR active photonics and therefore constitutes an important step toward expanding the mid-IR optics toolbox.

1. Architectural Novelty and Mechanism Translation
The authors heavily defend their novelty by claiming this is the "first use of high-Q silicon membranes for fast free-space mid-IR light modulation," attempting to distinguish their work from prior static SEIRA sensors. However, the fundamental paradigm of utilizing asymmetric, free-standing silicon nanostructures (breaking symmetry to excite high-Q modes) for active free-space spatial light modulation is already well-established. For instance, Kwon et al. (Nat. Commun. 13, 5811, 2022, DOI: 10.1038/s41467-022-33449-9) and Zheng et al. (Nano Lett. 23, 5588-5594, 2023, DOI: 10.1021/acs.nanolett.3c00999) have already demonstrated highly efficient amplitude and phase modulation using perturbed suspended silicon membranes without bottom mirrors.

Transitioning this exact structural paradigm from the near-infrared to the mid-infrared spectrum represents a solid engineering improvement through wavelength adaptation, rather than a fundamental breakthrough. In fact, scaling the operating wavelength into the mid-IR inherently relaxes the fabrication constraints. Because the critical feature sizes of the meta-atoms scale up proportionally with the longer wavelength, the lithographic resolution requirements are lowered, making the device fabrication physically easier and more forgiving. Coupling this up-scaled static structural paradigm with the classical tuning mechanisms of silicon (thermo-optic and free-carrier dispersion) does not establish a fundamentally new light-matter interaction regime warranting publication in a general-interest, high-impact journal.

Our response:
We thank the reviewer for the insightful comparison to prior suspended silicon metasurfaces. However, we stress again that translation of these concepts to the mid-IR is not a mere wavelength adaptation and design scaling. Transitioning to this regime drastically changes the extent of refractive modulation in silicon, in particular due to the relative strength and spectral position of free-carrier dispersion with respect to the optical resonance. This leads to a qualitatively different operational window, where strong carrier-induced index modulation, ultra-high-Q resonant enhancement, and low-fluence all-optical switching can be simultaneously exploited.

It is also important to reiterate that we demonstrate DUV-fabricated (device ready) ultra-high-Q resonances combined with large modulation depth and high-speed tuning within a single free-space platform. This represents a deliberate integration of favorable material properties into a distinct mid-IR operational regime, enabling performance beyond what is expected from simple scaling of existing NIR concepts.

2. The Overstatement of Mid-IR Scarcity and Speed Bottlenecks
The authors highlight a supposed scarcity of actively tunable mid-IR devices to justify their metrics. However, this assertion misrepresents the current state-of-the-art, which has already advanced to highly integrated, 2D-addressable architectures with superior speeds.

For instance, Popescu et al. (arXiv:2511.03583, 2025) have recently demonstrated a fully 2D-addressable mid-IR transmissive spatial light modulator utilizing CMOS-foundry microheaters and integrated PIN diode selectors. In comparison, the bulk interdigitated heating architecture presented in this manuscript is architecturally primitive and lacks true spatial addressability. Furthermore, regarding the authors' claim of "fast" electro-thermal tuning (14.5 kHz), recent works have vastly outperformed this metric. Liu et al. (Nat. Commun. 16:1665, 2025, DOI: 10.1038/s41467-025-56811-z) recently demonstrated electrically programmable mid-IR metasurfaces utilizing quasi-bound states in the continuum (QBIC)—the exact resonance physics claimed here—achieving electro-thermal modulation speeds of ~146 kHz. In light of these concurrent mid-IR advancements, the present manuscript's electro-thermal speed (14.5 kHz) represents an evolutionary technical upgrade within expected thermal constraints, rather than a forward-looking paradigm shift.

Our response:

We thank the Reviewer for highlighting these important recent developments in actively tunable mid-IR photonics. We agree that the field is progressing rapidly toward sophisticated integrated architectures, including 2D-addressable platforms and high-speed active metasurfaces.

We emphasize, however, that our work addresses a substantially different operating regime and set of design constraints than the examples cited by the Reviewer. We therefore view these works as complementary rather than competing approaches. Our focus is on transmissive free-space modulation, where we simultaneously achieve high absolute modulation depth, low optical loss, and compatibility with scalable fabrication.

For example, the relatively high switching speed demonstrated by Popescu et al. is enabled by efficient thermal sinking through the bulk substrate. At the same time, this architecture imposes substantial limitations on transmission efficiency, as explicitly noted by the authors themselves ("…while GSST offers broadband transparency in both phases extending beyond 20 μm, practical operation is constrained by absorption in the buried oxide and doped-silicon heater").

Similarly, solutions developed for reflective configurations, such as the architecture proposed by Liu et al., can benefit from thermal management conditions enabled by IR-opaque but thermally conductive substrates. In contrast, suspended silicon membranes operate in a fundamentally different regime, where thermal isolation simultaneously enhances modulation efficiency and limits thermal dissipation rates.

Furthermore, while switching speed is undoubtedly an important metric, it is not the only figure of merit for practical optical devices, particularly in light modulation and sensing applications. Modulation depth and related optical throughput are equally critical parameters, as discussed by Conteduca et al. (DOI: 10.1021/acsphotonics.2c00188). In this regard, the combination of large modulation amplitude with record-high Q-factors represents a distinctive advantage of our platform.

Actions:

We highlighted the importance of simultaneously high Q-factor and modulation amplitude in the revision:

...Notably, it combines a very large amplitude contrast close to 55% and a high quality factor of Q=2965, which are equally important for both sensing and light modulation applications [(DOI: 10.1021/acsphotonics.2c00188)]

3. Physical Instability and Artificial Testing Environment
With a measured Q-factor near 3000, the resonance linewidth is exceptionally narrow. The authors state that this sharp spectral slope is utilized to enhance modulation depth with low driving amplitude. However, the current demonstration is highly idealized and fragile.

To even capture this resonance, a custom FTIR accessory with extreme spatial filtering was required to choke the effective NA down to 0.02. In any real-world free-space mid-IR deployment (e.g., coupling with narrowband quantum cascade lasers or thermal emission management), light beams carry inherent angular divergence. Furthermore, even minor environmental thermal fluctuations or laser source frequency jitters will cause the bias point to drift completely off this narrow slope. Without demonstrating long-term operational stability or a robust feedback-locking mechanism, the functional benefit of this high-Q configuration in real-world applications remains unsubstantiated.

Our response:
We thank the reviewer for pointing out this important technical aspect. Small angular tolerance of the modulator indeed can limit the range of input optical signals. However, a quick calculation shows that NA of 0.02 is not restrictive and covers realistic use cases:

1. NA of 0.02 corresponds to a full angular divergence of 2.3 degrees (or 40 mrad). In turn, at a wavelength of 5 um, such angular divergence corresponds to diffraction of a mid-IR beam with a gaussian diameter of only 160 um (or equivalent Airy aperture of 300 um), which means that our membrane modulators

are capable of addressing the entire beam size without deterioration of their performance.

2. For a more realistic scenario of a light beam (e.g., QCL output) focused on the modulator, NA=0.02 corresponds to a standard beam with 2mm diameter focussed with a 50 mm lens.

We further argue that angular tolerance and stability is well controlled by the metasurface design.

Actions:
We added the above quantitative estimate of the effective angular acceptance in the revised manuscript, to put it into a practical perspective:
“This NA corresponds to a full angular divergence of approximately 2.3° (40 mrad), which remains compatible with typical mid-IR beam sizes and focusing conditions.”

4. The Illusion of System-Level Low Power Consumption
For the all-optical modulation route, the authors highlight that the required pump fluence is two orders of magnitude lower than that in the near-infrared or visible ranges. However, from a practical, system-level perspective, this scheme inherently requires an external, bulky, and power-hungry ultra-fast pulsed laser system to act as the pump control. Minimizing the energy absorption on the metasurface chip at the expense of introducing a high-overhead external optical setup does not translate to genuine "low-power integrated photonics." The overall system footprint severely undermines the claimed practical integration advantages.

Our response:

We thank the Reviewer for this important comment. We agree that the experimental use of an ultrafast pulsed laser system does not, by itself, represent a compact system-level implementation. However, we emphasize that the ultrafast optical setup in our work is primarily as a characterization tool for probing the ultimate carrier and thermal dynamics of the metasurface platform, rather than as a prerequisite component of the modulation concept itself.

Specifically, the femtosecond pump-probe configuration enables us to resolve the fastest physical processes governing the modulation bandwidth and carrier relaxation dynamics in suspended silicon membrane metasurfaces. At the same time, the demonstrated modulation mechanism is not fundamentally tied to the use of bulky ultrafast laser systems.

Our key observation is that the required absorbed optical fluence for inducing substantial modulation in the mid-IR regime is significantly reduced compared to similar implementations in the visible and near-IR spectral ranges. This result directly reflects the enhanced free-carrier effects in the mid-IR and represents an intrinsic property of the platform rather than a unique feature of the external excitation source.

We additionally note that externally driven optical modulation schemes are widely employed for studying and benchmarking ultrafast photonic devices, including systems ultimately intended for operation with more compact or application-specific excitation sources. In this context, the present work establishes the achievable modulation limits and identifies the crucial dynamic mechanisms in suspended mid-IR silicon metasurfaces.

Reviewer #2 (Remarks to the Author):

All my previous comments have been satisfactorily addressed. The only issue is that "HARPIA-TA" is not added in the revised manuscript.

Our response and Actions:
We thank the reviewer for the valuable feedback throughout the process. We also thank the reviewer for bringing up this point to our attention. The laser model was explicitly stated in the Methods section of the manuscript. Based on the comment, we realize that this information was not properly referenced in the main text, which we fixed in the latest revision.

Reviewer #3 (Remarks to the Author):

The revised manuscript addresses all my points. I recommend it for publication in Nature Communications.

Our response:
We thank the reviewer for the valuable suggestions and comments.

Reviewer #4 (Remarks to the Author):

I co-reviewed this manuscript with one of the reviewers who provided the listed reports. This is part of the Nature Communications initiative to facilitate training in peer review and to provide appropriate recognition for Early Career Researchers who co-review manuscripts.

Our response:
We thank the reviewer for the valuable contribution to the review process.
